# Association between Genotype and the Glycemic Response to an Oral Glucose Tolerance Test: A Systematic Review

**DOI:** 10.3390/nu15071695

**Published:** 2023-03-30

**Authors:** Sandra Bayer, Anna Reik, Lena von Hesler, Hans Hauner, Christina Holzapfel

**Affiliations:** 1 Institute for Nutritional Medicine, School of Medicine, University Hospital “Klinikum Rechts der Isar”, Technical University of Munich, 80992 Munich, Germany; 2 Else Kröner-Fresenius-Center for Nutritional Medicine, School of Life Sciences, Technical University of Munich, 85354 Freising, Germany; 3 Department of Nutritional, Food and Consumer Sciences, Fulda University of Applied Sciences, 36037 Fulda, Germany

**Keywords:** gene, genetic, metabolic, nutrition, postprandial

## Abstract

The inter-individual variability of metabolic response to foods may be partly due to genetic variation. This systematic review aims to assess the associations between genetic variants and glucose response to an oral glucose tolerance test (OGTT). Three databases (PubMed, Web of Science, Embase) were searched for keywords in the field of genetics, OGTT, and metabolic response (PROSPERO: CRD42021231203). Inclusion criteria were available data on single nucleotide polymorphisms (SNPs) and glucose area under the curve (gAUC) in a healthy study cohort. In total, 33,219 records were identified, of which 139 reports met the inclusion criteria. This narrative synthesis focused on 49 reports describing gene loci for which several reports were available. An association between SNPs and the gAUC was described for 13 gene loci with 53 different SNPs. Three gene loci were mostly investigated: *transcription factor 7 like 2* (*TCF7L2*), *peroxisome proliferator-activated receptor gamma* (*PPARγ*), and *potassium inwardly rectifying channel subfamily J member 11* (*KCNJ11*). In most reports, the associations were not significant or single findings were not replicated. No robust evidence for an association between SNPs and gAUC after an OGTT in healthy persons was found across the identified studies. Future studies should investigate the effect of polygenic risk scores on postprandial glucose levels.

## 1. Introduction

It is well established that the postprandial response to standardized meals shows high inter-individual variability [1,2,3]. Health status, genotype, medication, dietary habits, lifestyle factors, and other phenotypic features (e.g., stress, sleep) are possible contributors to this variability [1,2,3,4].

Genome-wide association studies (GWAS) have identified associations between single nucleotide polymorphisms (SNPs) and fasting glucose levels. For instance, the Meta-Analysis of the Glucose and Insulin-related traits Consortium (MAGIC) reported several independent genetic loci associated with glucose metabolism [5]. Furthermore, a meta-analysis of nine GWAS, with 15,234 participants without type 2 diabetes mellitus (T2DM), revealed five genetic loci that are associated with the 2-hour glucose level after an oral glucose tolerance test (OGTT) [6], indicating that SNPs also affect postprandial glucose metabolism. However, Berry et al. (2020) have recently shown that genotypes play a minor role as predictors of the postprandial response to a standardized meal challenge [1].

The postprandial 2-hour glucose level is frequently used as a clinical parameter for the classification of disturbances of glucose metabolism and is of diagnostic value for T2DM. In this study, we focus on the glucose area under the curve (gAUC) as the primary outcome as an approximation of glucose metabolism and evaluate the genetic contribution to its variability in healthy persons. In the emerging research of precision nutrition, there is growing interest in detecting genotype–phenotype interactions that may explain inter-individual variations since this information might be promising for individualized dietary recommendations [7].

Therefore, we performed a systematic review to obtain an overview of current research on the associations between SNPs of any gene loci and the glycemic response to an OGTT, calculated as gAUC, in persons without diabetes.

## 2. Methods

This review is registered in the International Prospective Register for Systematic Reviews (PROSPERO, registration number CRD42021231203) and follows the Preferred Reporting Items for Systematic Review and Meta-Analyses protocol [8].

### 2.1. Search Strategy

Starting in January 2021, three electronic databases (Web of Science, Embase, PubMed) were searched for records meeting the following search items based on three blocks: genetics, intervention, and outcome. In the first block, search items were “polymorphism”, “polymorphisms”, “genotype”, “genotypes”, “variant”, “variants”, “SNP”, “SNPs”, “gene locus”, “gene loci”, “genetic locus”, and “genetic loci”. The following search items were used for the intervention block: “OGTT”, “challenge”, “challenges”, “oral”, “hour”, “tolerance test”, “tolerance tests”, “fasting”, and “glucose tolerance”. The third block included the following search items: “glucose”, “glycemic”, “glycaemic”, “postprandial”, “response”, and “responses”. The Boolean “OR” was used to combine search items within each block, while the Boolean “AND” was applied to combine the three blocks. Depending on the database, filters for language (English), species (Human), and the publication year (since 2000) were applied. For the identification of additional records, the reference list of eligible reports was checked by hand.

### 2.2. Study Selection

The study selection followed the PICO (population, intervention, control, and outcomes) criteria [9]. The requirements for inclusion were: (a) availability of SNP data, (b) intervention: OGTT, (c) outcome: calculated as gAUC, and (d) association between SNP and gAUC. Reports were excluded if: (a) language was not English, (b) animal or plant studies, and (c) special cohort characteristics (e.g., severe disease, pregnant/breastfeeding women, children, participants with diabetes). Studies in persons with diabetes were included in this narrative review if statistical analyses of interest have been performed in a subgroup of persons without diabetes. The review team consisted of four reviewers (S.B., A.R., L.v.H., C.H.). S.B. and L.v.H. independently screened titles, abstracts, and full texts for eligibility. In case of discrepant evaluations, A.R. and C.H. assessed the reports for eligibility. Authors were contacted in case of missing full text. The screening organization process was done by Microsoft Excel 2016 (Microsoft Corp, Redmond, WA, USA) and by the reference management software EndNote X9 (Thomsen Reuters, New York, NY, USA).

### 2.3. Data Extraction

Two reviewers (S.B., L.v.H.) independently extracted the following data to an Excel sheet: authors, publication year, study name, description of the study population, the sample size for gAUC calculation, intervention time, genes of interest, SNPs, statistical results, and details. For the calculation of the linkage disequilibrium (LD), the genome browser Ensembl was used [10].

### 2.4. Reporting Strategy

This review treated all reports based on their statistical results equally. A narrative synthesis was used to present and summarize data. According to the PROSPERO registration, no meta-analysis was performed.

### 2.5. Quality Assessment

The assessment tool for the quality evaluation of genetic association studies, according to Campell and Rudan, was applied [11]. Eleven questions on chance, risk, and confounding factors were answered to describe the validity of associations between SNP and gAUC. The rating was done as follows: rather high quality (5.5 to 11 points), intermediate quality (0 to 5 points), or low quality (−11 to −0.5 points). The rating was according to the author’s opinion as Campell and Rudan stated no information about the rating procedure [11]. Since the calculation of associations between SNPs and gAUC was mostly performed as a post-hoc analysis, no risk of bias assessment occurred.

## 3. Results

**Figure 1:**
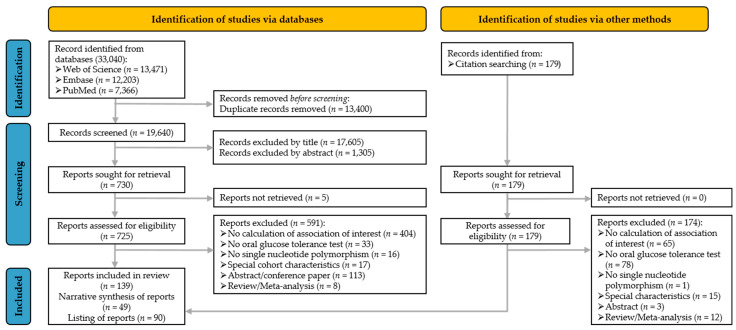
Flow chart of the systematic literature search according to [8].

The search in three electronic databases provided 33,040 records, of whom 13,400 records were removed as duplicates and a further 18,910 records were excluded during the title and abstract screening (Figure 1). For the full-text screening, 17 authors were contacted to provide more information regarding their articles, out of whom 12 authors provided the missing full texts. In total, 139 reports matched the PICO criteria. In those, associations between 96 gene loci and the gAUC after an OGTT were assessed. 

In this narrative synthesis, gene loci were included, for which at least three reports were available (49 reports) (Figure 1). This restriction of gene loci was crucial to increase the informative value and to reduce the presentation of single, not-replicated findings. Information on gene loci, for which one (68 gene loci) or two reports (15 gene loci) were available, are presented in Supplementary Tables S1 and S2.

### 3.1. Characteristics of the Identified Studies

A total of 49 eligible reports investigated the association between SNPs and the gAUC after an OGTT in 39 different cohorts, i.e., Quebec Family Study [12,13,14,15,16,17], Amish Family Diabetes Study [18,19], Ely Study [20], Tübingen Family Study (TÜF) [21], European Atherosclerosis Research Study (EARS II) [22,23], Sapphire Study [24], Metabolic Intervention Cohort Kiel (MICK) [25,26], European Network on Functional Genomics of Type 2 Diabetes (EUGENE2) [27,28], Metabolic Syndrome in Men (METSIM) [28], Metabolic Syndrome Berlin Potsdam Study (MESYBEPO) [29], Lifestyle Intervention in a General Population for Prevention of Ischaemic Heart Disease (Inter99) [30,31], Berlin Ernährung Geschwister Study (BErG-Study) [32], and further 27 cohorts without a specific study name [33,34,35,36,37,38,39,40,41,42,43,44,45,46,47,48,49,50,51,52,53,54,55,56,57,58,59,60] (Table 1, Table 2, Table 3 and Table 4).

Most cohorts included participants of European descent. French-Canadian or African American participants were included in either one or two cohorts, whereas four other cohorts included participants from Asia. Reports were published between 2000 and 2020. The sample size ranged from 18 to 4430 participants. In most studies, a standardized OGTT with 75 g glucose was conducted. In one study, a glucose amount of either 50 or 75 g was used without any justification [42]. While in most of the reports, the duration of the OGTT lasted 120 min [20,21,22,23,24,27,28,29,30,31,32,33,36,37,38,39,41,43,44,45,47,48,50,52,53,54,56,57,58,59,60] or 180 min [12,13,14,15,16,17,18,19,35,40,42,49,51,55], in either two reports the OGTT was performed over 240 min [25,26] or 300 min [34,46].

### 3.2. Study Quality Assessment

**Figure 2:**
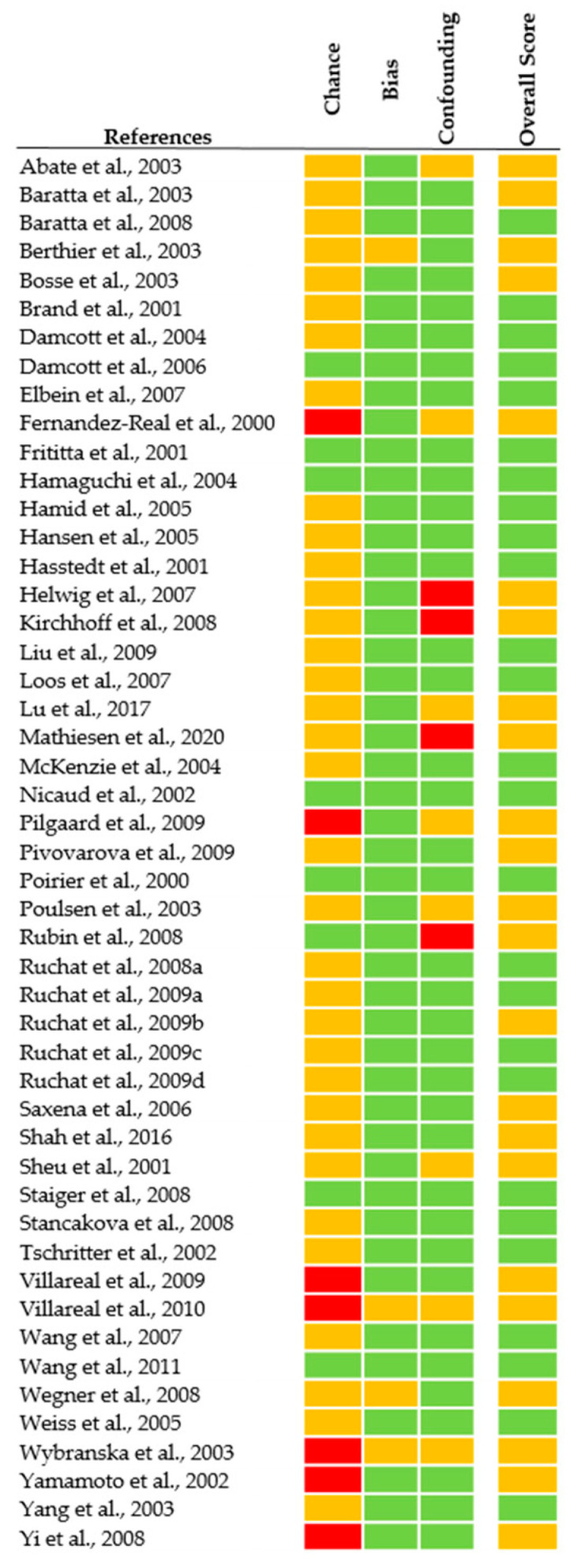
Quality assessment of genetic association studies [11]. The quality ratio was rather high (green), intermediate (yellow), or low (red). Abate et al. 2003 [49]; Baratta et al. 2003 [37]; Baratta et al. 2008 [50]; Berthier et al. 2003 [51]; Bosse et al. 2003 [14]; Brand et al. 2001 [32]; Damcott et al. 2004 [19]; Damcott et al. 2006 [18]; Elbein et al. 2007 [58]; Fernandez-Real et al. 2000 [52]; Frititta et al. 2001 [53]; Hamaguchi et al. 2004 [54]; Hamid et al. 2005 [30]; Hansen et al. 2005 [31]; Hasstedt et al. 2001 [59]; Helwig et al. 2007 [25]; Kirchhoff et al. 2008 [21]; Liu et al. 2009 [60]; Loos et al. 2007 [20]; Lu et al., 2017 [41]; Mathiesen et al., 2020 [42]; McKenzie et al., 2004 [55]; Nicaud et al., 2002 [23]; Pilgaard et al., 2009 [43]; Pivovarova et al., 2009 [29]; Poirier et al., 2000 [22]; Poulsen et al., 2003 [39]; Rubin et al., 2008 [26]; Ruchat et al., 2008a [16]; Ruchat et al., 2009a [17]; Ruchat et al., 2009b [12]; Ruchat et al., 2009c [15]; Ruchat et al., 2009d [13]; Saxena et al., 2006 [44]; Shah et al., 2016 [45]; Sheu et al., 2001 [56]; Staiger et al., 2008 [27]; Stancakova et al., 2008 [28]; Tschritter et al., 2002 [33]; Villareal et al., 2009 [34]; Villareal et al., 2010 [46]; Wang et al., 2007 [47]; Wang et al., 2011 [35]; Wegner et al., 2008 [48]; Weiss et al., 2005 [40]; Wybranska et al., 2003 [57]; Yamamoto et al., 2002 [38]; Yang et al., 2003 [24]; Yi et al., 2008 [36].

The results of the quality assessment are shown in Figure 2. No report was rated as low quality. The quality of 23 reports was judged to be intermediate, since information on the power calculation, correction for multiple testing, adjustment, and/or ethnicity was missing. The remaining 26 studies were rated as high quality (Figure 2).

### 3.3. Main Findings

In total, the association between SNPs and gAUC after an OGTT was assessed for 13 genes and 53 different SNPs. The most frequently examined genes included *transcription factor 7 like 2* (*TCF7L2*) with 15 eligible reports (Table 1) [12,13,18,20,21,41,42,43,44,45,46,47,48,58,60], followed by *peroxisome proliferator-activated receptor gamma* (*PPARγ*) with ten reports (Table 2) [14,15,22,24,25,37,38,39,40,59], and *potassium inwardly rectifying channel subfamily J member 11* (*KCNJ11*) with six reports (Table 3) [12,13,33,34,35,36]. Furthermore, the following gene loci have been investigated in three to five reports: *adiponectin* (*ADIPOQ*) [16,24,26], *CDK5 regulatory subunit-associated protein 1-like 1* (*CDKAL1*) [12,13,21,28], *cyclin-dependent kinase inhibitor-2A/B* (*CDKN2A/2B*) [12,13,21], *hematopoietically expressed homeobox* (*HHEX*) [12,13,21,27,29], *hepatocyte nuclear factor 4α* (*HNF4α*) [17,19,31], *insulin-like growth factor 2 mRNA-binding protein 2* (*IGF2BP2*) [12,13,21], *interleukin-6* (*IL6*) [30,51,52,55], *prohormone convertase 1* (*PC-1*) [37,49,50,53,54], *solute carrier family 30 zinc transporter member 8* (*SLC30A8*) [12,13,21], and *tumor necrosis factor-alpha* (*TNF-α*) (Table 4) [23,32,56,57].

#### 3.3.1. *Transcription Factor 7 Like 2 (TCF7L2)*

An association between the *TCF7L2* gene locus and gAUC after an OGTT was examined in 15 different cohorts (Table 1) [12,13,18,20,21,41,42,43,44,45,46,47,48,58,60]. Most of the 17 SNPs within the *TCF7L2* gene locus were investigated in one (13 SNPs) or two cohorts (2 SNPs), respectively. 

The association between the SNP rs12255372 and the gAUC was investigated in five different cohorts (Table 1) [18,20,47,58]. Homozygous carriers of the minor allele (T) showed a significantly higher gAUC compared to heterozygous carriers and the wild-type (*p* = 0.04) in 1697 participants from the Ely study [20]. Similar results were found in 1538 Finnish men, where homozygous and heterozygous carriers of the minor allele (T) showed a higher gAUC than the wild-type (*p* = 0.039) [47]. These results could not be replicated in the cohort of the Amish Family Diabetes Study [18], the non-diabetic offsprings of persons with T2DM [47], or participants without a family history of T2DM (*p* > 0.05) [58].

The SNP rs7903146, which is in high LD (r^2^ > 0.8) with the SNP rs12255372, was examined in different cohorts (Table 1) [12,13,18,20,21,41,42,43,44,45,46,48,58]. While in eight cohorts, no significant difference between the genotypes and gAUC was found [12,13,18,42,43,44,46,58], there was a statistically significant difference between the genotypes in two cohorts. In the Ely study, homozygous carriers of the minor allele (T) showed a significantly higher gAUC compared to heterozygous carriers and the wild-type (*p* = 0.013) [20]. A significantdifference was found between homozygous and heterozygous carriers of the minor allele (T) compared to the wild-type in 1065 participants of the TÜF cohort (*p* = 0.001) [21]. In two cohorts, the results for an association between this SNP and gAUC were inconsistent, depending on the selection of participants or the calculation method of the gAUC [41,45,48]. In the first cohort of 120 persons without diabetes, homozygous carriers of the minor allele (T) had a significantly higher gAUC than the wild-type [41,45]. A similar result was found for women (*p* < 0.05), while no association was found for men (*p* > 0.05) [41]. In the second cohort, carriers of the minor allele (T) had a significantly higher gAUC compared to the wild-type (Table 1) [48].

#### 3.3.2. *Peroxisome Proliferator-Activated Receptor Gamma (PPARγ)*

Associations between the *PPARγ* gene locus, SNP rs1801282, and the gAUC were analyzed in nine cohorts (Table 2) [14,15,22,24,25,37,38,39,40,59]. No significant differences between specific genotypes were found in several cohorts (Table 2) [14,15,22,25,37,38,40,59]. In 1713 participants from the Sapphire Study, homozygous and heterozygous carriers of the minor allele (G) showed a significantly different gAUC after an OGTT (*p* = 0.0210) [24]. In a cohort with 549 elderly Danish homozygotic twins, carriers of the minor allele (G) had a higher gAUC during OGTT compared to the wild-type (*p* = 0.016) [39]. However, these results could not be replicated in a subgroup analysis with 54 dizygotic twin pairs (*p* = 0.19) [39].

#### 3.3.3. *Potassium Inwardly Rectifying Channel Subfamily J Member 11 (KCNJ11)*

The *KCNJ11* gene locus was examined for an association with gAUC after an OGTT in six cohorts (Table 3) [12,13,33,34,35,36]. Within the *KCNJ11* gene locus, five SNPs were investigated, of whom three SNPs were examined in one cohort [12,13].

In a cohort of Han-Chinese participants, the findings were inconsistent, depending on the included participants or the genetic model [35]. While a significant difference between the genotypes in 667 normoglycemic participants was found in the additive (*p* = 0.006) and dominant (*p* = 0.007) models, no difference was observed for the gAUC between the genotypes in the recessive model. However, the significance disappeared after the correction for multiple testing. Independent of the genetic model, no significant association between SNP rs5215 and the gAUC was found in 458 participants with impaired glucose tolerance and impaired fasting glucose [35]. No association was found between genotypes and the gAUC in 669 participants from the Quebec Family Study (Table 3) [13].

An association between SNP rs5219, which is in high LD (r^2^ > 0.8) with SNP rs5215, and the glycemic response to glucose was investigated in five cohorts [33,34,35,36]. No significant difference in the gAUC was observed in four cohorts [34,35,36]. In 298 persons without diabetes, carriers of the minor allele (T) had an increased gAUC compared to the wild-type when using the dominant genetic model (*p* = 0.04) or by comparing homozygous carriers of the minor allele with the wild-type (*p* = 0.02) [33]. No significant difference was seen when using the additive model *(p* = 0.05) [33]. In a subgroup analysis of 75 persons who underwent an OGTT and, in addition, a hyperglycemic clamp, the dominant model resulted in a significantly increased gAUC in carriers of the minor allele (T) compared to the wild-type (*p* = 0.02) (Table 3) [33].

#### 3.3.4. Further Genes

Findings for further genes are presented in Table 4. The association between four SNPs within the *CDKAL1* gene locus and the gAUC was assessed in four cohorts (Table 4) [12,13,21,28]. For the most examined SNP rs7754840, a significant difference in the gAUC was found between homozygous and heterozygous carriers of the minor allele (C) and the wild-type in 846 participants from the EUGENE2 study (*p* = 0.016) [28]. Similar findings were found for 1065 participants from the TÜF cohort (*p* = 0.02) [21], while no significant difference between the genotypes and the gAUC was found for 3367 participants without diabetes from the METSIM cohort [28]. In the Quebec Family Study with 669 participants, the rs7756992, which is in a high LD (r^2^ > 0.8) with the SNP rs7754840, was not associated with the gAUC [13].

An association between the *HNF4α* gene locus and the glucose response was studied in three cohorts (Table 4) [17,19,31]. Out of six SNPs, four SNPs were investigated in one cohort and showed no significant association [17,19,31]. SNP rs1884614 was examined in 689 participants from the Amish Family Diabetes Study [19] and 4430 participants from the Inter99 Study [31]. In both cohorts, homozygous and heterozygous carriers of the minor allele (T) showed significantly different gAUC than the wild-type. While a significant difference was seen in the Amish population with the additive genetic model (*p* = 0.022) [19], no difference was seen in the Danish cohort in the additive (*p* = 0.05) as well as in the recessive genetic model (*p* = 0.21) [31]. Associations between SNP rs1885088 and the gAUC were investigated in the Inter99 Study [31] as well as in the Quebec Family Study [17]. In both cohorts, no significant difference was observed between the genotypes. In a sub-analysis within the Quebec Family Study, homozygous carriers of the minor allele (A) with a high physical activity level showed significantdifferences in the gAUC than heterozygous carriers (*p* = 0.01) or the wild-type (*p* = 0.01) [17]. No association was detected in participants with a low physical activity level (Table 4) [17].

## 4. Discussion

In 139 reports, 96 different gene loci were investigated for an association with gAUC after an OGTT. This narrative synthesis included 49 reports [12,13,14,15,16,17,18,19,20,21,22,23,24,25,26,27,28,29,30,31,32,33,34,35,36,37,38,39,40,41,42,43,44,45,46,47,48,49,50,51,52,53,54,55,56,57,58,59,60] in which one specific gene was assessed in at least three separate reports. Overall, the results for the most frequently investigated genetic loci *(TCF7L2, PPARγ, KCNJ11, ADIPOQ, CDKAL1, CDKN2A/B, HHEX, HNF4α, IGF2BP2, IL-6, PC-1, SLC30A8, TNF-α)* were heterogeneous [12,13,14,15,16,17,18,19,20,21,22,23,24,25,26,27,28,29,30,31,32,33,34,35,36,37,41,42,43,44,45,46,47,48,49,50,51,52,53,54,55,56,57,58,59,60].

Most reports investigated an association between *TCF7L2* SNPs (rs12255372 and rs7903146, LD r2 > 0.8) and the gAUC [12,13,18,20,21,41,42,43,44,45,46,47,48,58,60]. For both SNPs, reports based on the biggest sample sizes (SNP rs12255372: Ely study: 1697 participants [20], 1538 Finnish men [47], SNP rs7903146: Ely study: 1697 participants [20], TÜF cohort: 1065 participants [21]) found a significantly higher gAUC in carriers of the minor allele (T) compared to heterozygous carriers and/or the wild-type. However, for the TÜF cohort, no information about any statistical adjustment was given [21]. In contrast, no statistical significance was found in most of the smaller cohorts, including sample sizes between 18 and 721 participants [12,13,18,21,41,42,43,44,45,46,47,48,58,60]. These results indicate that the SNPs rs12255372 and rs7903146 may modify the gAUC after an OGTT. However, false-positive results cannot be excluded since the statistical power to detect significant associations between the SNPs and gAUC is unknown. There is some evidence from GWAS, that were excluded from this narrative synthesis, that the *TCF7L2* gene locus influences glucose metabolism not only in the fasting state [6,61] but also in the post-challenge phase [6]. A meta-analysis of several GWAS, including 15,234 participants without diabetes, showed that the SNP rs7903146 was associated with fasting glucose and 2-h glucose level after an OGTT [6]. However, no association could be found between the SNP rs7903146 and the AUC ratio of insulin to glucose [6].

Similar findings were found for an association between the *PPARγ* SNP rs1801282 and the gAUC [14,15,22,24,25,37,38,39,40,59]. For example, in the Sapphire cohort with 1713 participants, significant differences were found when comparing homozygous and heterozygous carriers of the minor allele (G) and the wild-type [24]. Nevertheless, in most cohorts, no significant association between rs1801282 and gAUC was found, possibly due to small sample sizes or different ethnicities. A meta-analysis with around 32,000 participants without diabetes revealed no evidence for an association between SNP rs1801282 and the 2-h glucose level; however, data on gAUC were not reported [62]. In addition, this meta-analysis revealed an association between the SNP and fasting glucose in participants with obesity [62]. To the best of our knowledge, there is no evidence so far for an association focusing on postprandial glucose trajectories. 

All analyses investigating the association between *KCNJ11* SNPs and gAUC were based on cohorts with less than 1000 participants [12,13,33,34,35,36]. For the most frequently assessed SNP rs5219, one report with 298 participants stated that carriers of the minor allele (T) had an increased gAUC compared to the wild-type [33]. However, the significance disappeared in the additive genetic model. Considering other weaknesses such as low sample sizes, different ethnicities, and missing correction for multiple testing, there is little evidence for a clinically relevant association between SNPs rs5215 or rs5219, and differences in gAUC after an OGTT. In addition, no data from GWAS for an association between the *KCNJ11* gene locus and gAUC are available. 

The eligible articles included data from the glucose response after a standardized 75 g OGTT in participants without diabetes. Potential confounding factors, e.g., age and BMI, were not considered mandatory for inclusion in this systematic review. Nevertheless, reports investigating the association between SNPs in the *TCF7L2*, *PPARγ,* as well as *KNCJ11* gene loci and the gAUC were based on participants with a BMI less than 30 kg/m^2^. Furthermore, most of the identified articles considered potential confounders in the adjustment procedure. However, the following differences between reports were obvious: frequency of plasma glucose measurement during the OGTT (every 10 min up to every hour), duration of the OGTT (120 min up to 300 min), sample size, ethnicity, and statistical methods (genetic model, adjustment, power calculation, and correction for multiple testing). Thus, the comparability between eligible reports might be limited not only by the high variability of SNPs investigated but also by these confounders. 

Several explanations for the given negative findings exist: firstly, the missing power to detect small effect differences among the genotypes. To detect small genetic effects on the metabolic response, cohorts with large sample sizes are needed. This was the reason for the establishment of large international consortia, namely, to be able to combine genetic data for the identification of SNPs with rather small effect sizes [63,64]. Out of the 39 different cohorts identified in our analysis, only 4 cohorts were found with a sample size above 1000 participants, which is not comparable to genetic association studies with more than, e.g., 35,000 persons [63]. Nevertheless, GWAS investigating the association between SNPs and the gAUC after an OGTT could not be identified, whereas data on GWAS regarding the association with 2-h postprandial glucose levels are frequently found [6,65].

Secondly, other factors with a greater effect on gAUC might have masked any genetic effect. The Personalized Responses to Dietary Composition (PREDICT) study revealed that factors such as meal composition have a greater effect on the gAUC after a meal challenge than the genotype (15.4% vs. 9.5%) [1]. Addiotionally, the assessment of the association between SNPs and gAUC after an OGTT was not the primary aim of most studies, and usually, a post-hoc analysis was performed. Moreover, due to the missing clinical endpoint of the gAUC, the clinical relevance of the investigated association is difficult to determine. 

Furthermore, the most frequently studied gene loci, *TCF7L2* [66,67,68], *PPARγ* [69,70], and *KCNJ11* [71,72] are candidate genes for T2DM predisposition. This hypothesis-driven approach, with identified candidate genes, turned out to be of limited value in predicting people with early disturbances in glucose metabolism. It is rather likely that other gene loci or combinations thereof may also play a role for the metabolic response after an OGTT. The *gastric inhibitory polypeptide receptor* (*GIPR)* gene locus is one of the known genes to affect the metabolic response after an OGTT [6]. The *GIPR* SNP rs10423928 was associated with the 2-h glucose level and the AUC ratio of insulin and glucose after an OGTT in participants without diabetes [6]. However, the association between the *GIPR* gene locus and the gAUC could not be identified in any eligible article of this systematic review. 

Finally, no main single effect of an SNP on gAUC after an OGTT was found. Therefore, it may be worthwhile to study the effect of a combination of SNPs. In several studies, the association between a polygenetic risk score and gAUC after an OGTT was analyzed [73,74,75,76,77,78]. Depending on the chosen gene loci for the calculation of a risk score, both significant [73,76,77] and non-significant [24,74,75,78] differences were found for gAUC per risk allele. Therefore, research on polygenic risk scores might be more meaningful to evaluate a genetic effect on the metabolic response after an OGTT. So far, most candidate genes for T2DM or gene loci known to interfere with glucose metabolism were used for the calculation of the genetic risk score [73,74,75,76,77,78]. Machine learning approaches and artificial intelligence measures open further possibilities for a more comprehensive understanding of the genetic contribution to metabolic responses after an OGTT. Genome-wide polygenic risk scores may be even more promising in this context [79].

### Strengths and Limitations

This systematic review focused on OGTT as the standard method to characterize glucose metabolism. For all included reports, the methodological quality of genetic associations was assessed and presented. This systematic review is limited by focusing on SNPs and by excluding other genetic variants such as copy number variations and haplotypes. Findings are based on hypothesis-driven approaches, including candidate genes. As the gAUC is not a clinical parameter with a defined diagnostic or clinical value, no assessment of the clinical effect can be made. Furthermore, in most of the included cohort studies, the performance of the OGTT was for the classification of participants according to their glucose metabolism, e.g., normoglycemic or diabetic, rather than on the primary or secondary outcomes. This systematic review is focused on persons without diabetes to address the research gap on the association between SNPs and metabolic response on an OGTT in healthy persons to follow the current discussion on the inter-individual variation of metabolic response in a standardized meal challenge as a predictor for personalized nutritional recommendations. Therefore, the considered sample sizes are rather small and a conclusion on gender-specific results was not possible. A narrative synthesis, as indicated in PROSPERO, was conducted since data pooling and performing a meta-analysis were not considered to be appropriate.

## 5. Conclusions

In this systematic review, which is based on candidate gene analyses, heterogeneous findings for the association between SNPs and the gAUC after an OGTT in participants without diabetes were detected. The most investigated genetic loci (*TCF7L2*, *PPARγ*, and *KCNJ11*) are known to increase the risk of developing T2DM and have shown single findings for a significant association with gAUC. Therefore, more robust data, including data from hypothesis-free approaches, are needed to exploit the genetic contribution to personalized nutrition.

## Figures and Tables

**Table 1:** Associations between *transcription factor 7 like 2* (*TCF7L2*) SNPs and gAUC after an OGTT in adults.

Study Characteristics	SNP	Sample Size Used	Results (*p*-Value) ^+^	Reference
**Quebec Family Study** French-Canadian families (phase 1: randomly selected; phase 2/3: at least one person with obesity per family) living in and around the Quebec city area	rs10128255	669 Participants without diabetes or treatment-naive participants	n. s. ^1,7,13^	[13]
	rs11196203		n. s. ^1,7,13^	
	rs11196205	669 Participants without diabetes or treatment-naive participants	n. s. ^1,7,13^	
**Amish Family Diabetes Study** Old Order Amish population, participants with previously diagnosed T2DM and first- and second-degree relatives, aged ≥ 18 years		661 Participants without diabetes	0.27 ^1,8^	[18]
**Quebec Family Study** French-Canadian families (phase 1: randomly selected; phase 2/3: at least one person with obesity per family) living in and around the Quebec city area	rs11594610 *	669 Participants without diabetes or treatment-naive participants	n. s. ^1,7,13^	[13]
**Ely Study** Ethnically homogenous Europid population without diabetes, aged 35–79 years	rs12243326 ^#^	1697 Participants without diabetes or treatment-naive participants	0.02 ^2,9^ Significantly higher gAUC in homozygous carriers of the minor allele (T) compared to heterozygous carriers and the wild-type	[20]
	rs12255372 ^#^	1697 Participants without diabetes or treatment-naive participants	0.04 ^2,9^ Significantly higher gAUC in homozygous carriers of the minor allele (T) compared to heterozygous carriers and the wild-type	
**Amish Family Diabetes Study** Old Order Amish population, participants with previously diagnosed T2DM and first- and second-degree relatives, aged ≥ 18 years		661 Participants without diabetes	0.92 ^1,8^	[18]
Population-based cross-sectional study with Finnish men, aged 50–70 years		1538 Participants without diabetes	0.039 ^3,10^ Homozygous and heterozygous carriers of the minor allele (T) showed higher gAUC compared to the wild-type	[47]
Non-diabetic offspring of patients with T2DM from Finland		238 Participants without diabetes	0.754 ^3,8^	
Participants of European and African American descent, cases with a first-degree relative with T2DM, and normoglycemic controls with no family history of T2DM		337 Europeans without diabetes	0.14 ^1,9^	[58]
		144 African Americans without diabetes	n. s. ^1,9^	
**Quebec Family Study** French-Canadian families (phase 1: randomly selected; phase 2/3: at least one person with obesity per family) living in and around the Quebec city area	rs12573128	653 Participants without diabetes or treatment-naive participants	0.009 ^1,7,13^ Significant difference between homozygous and heterozygous carriers of the minor allele (T) and the wild-type	[13]
	rs176632	669 Participants without diabetes or treatment-naive participants	n. s. ^1,7,13^	
	rs17685538		n. s. ^1,7,13^	
	rs1885510 *		n. s. ^1,7,13^	
Healthy, normotensive Caucasians without diabetes	rs290487	116 Participants without diabetes	0.62 ^1,9^	[60]
**Quebec Family Study** French-Canadian families (phase 1: randomly selected; phase 2/3: at least one person with obesity per family) living in and around the Quebec city area	rs3750804	669 Participants without diabetes or treatment-naive participants	n. s. ^1,7,13^	[13]
	rs3750805	653 Participants without diabetes or treatment-naive participants	0.02 ^1,7,13^ Significant difference between homozygous and heterozygous carriers of the minor allele (A) and the wild-type	
**Ely Study** Ethnically homogenous Europid population without diabetes, aged 35–79 years	rs4506565 ^$^	1697 Participants without diabetes or treatment-naive participants	0.003 ^2,9^ Significantly higher gAUC in homozygous carriers of the minor allele (T) compared to heterozygous carriers and the wild-type	[20]
**Quebec Family Study** French-Canadian families (phase 1: randomly selected; phase 2/3: at least one person with obesity per family) living in and around the Quebec city area	rs4918789	669 Participants without diabetes or treatment-naive participants	n. s. ^1,7,13^	[13]
	rs7901695 ^$^	669 Participants without diabetes or treatment-naive participants	n. s. ^1,7,13^	
		712 Participants without diabetes or treatment-naive participants	n. s. ^1,7,13^	[12]
**Amish Family Diabetes Study** Old Order Amish population, participants with previously diagnosed T2DM and first- and second-degree relatives, aged ≥ 18 years		683 Participants without diabetes	0.82 ^1,8^	[18]
**Quebec Family Study** French-Canadian families (phase 1: randomly selected; phase 2/3: at least one person with obesity per family) living in and around the Quebec city area	rs7903146 ^#,$^	669 Participants without diabetes or treatment-naive participants	n. s. ^1,7,13^	[13]
		712 Participants without diabetes or treatment-naive participants	n. s. ^1,7,13^	[12]
Different family-based and case–control studies from Europe and U.S.		721 Participants without diabetes	n. s. ^1,10^	[44]
			n. s. ^2,10^	
			n. s. ^4,10^	
			n. s. ^5,10^	
			n. s. ^6,10^	
Participants without diabetes, aged 20–70 years, randomly selected from the area around Mayo Clinic Rochester		120 Participants without diabetes	<0.01 ^4,9^ Significantly higher gAUC in homozygous carriers of the minor allele (T) compared to the wild-type	[41]
		45 Men without diabetes	n. s.^4,9^	
Participants without diabetes, aged 20–70 years, randomly selected from the area around Mayo Clinic Rochester	rs7903146 ^#,$^	75 Women without diabetes	<0.05 ^8,13^ Significantly higher gAUC in homozygous carriers of the minor allele (T) compared to the wild-type	[41]
		120 Participants without diabetes	0.003 ^4,9^ Significantly higher gAUC in homozygous carriers of the minor allele (T) compared to the wild-type	[45]
**Ely Study** Ethnically homogenous Europid population without diabetes, aged 35–79 years		1676 Participants without diabetes or treatment-naive participants	0.013 ^2,9^ Significantly higher gAUC in homozygous carriers of the minor allele (T) compared to heterozygous carriers and the wild-type	[20]
		1537 Participants without diabetes	< 0.05 ^2,9^ Significantly higher gAUC in homozygous carriers of the minor allele (T) compared to heterozygous carriers and the wild-type	
**Amish Family Diabetes Study** Old Order Amish population, participants with previously diagnosed T2DM and first- and second-degree relatives, aged ≥18 years		664 Participants without diabetes	0.28 ^1,8^	[18]
**TÜF** German participants with an increased risk of diabetes		1065 Participants without diabetes	0.001 ^1,11^ Significant higher gAUC in carriers of the minor allele (T) compared to heterozygous carriers and the wild-type	[21]
Population-based study, elderly same-sex Danish twins, Caucasian descent		531 Participants without diabetes or treatment-naive participants	AUC_0–120_: 0.006 ^1,7,14^ Significantly higher gAUC in carriers of the minor allele (T) compared to the wild-type	[48]
			AUC_0–30_: 0.2 ^1,7,14^	
Danish monozygotic and dizygotic twins (young and elderly) without diabetes		190 Participants without diabetes	n. s. ^1,7,14^	
Unrelated Caucasians (13%) and African American (5%) without diabetes and no family history of T2DM		18 Participants without diabetes	0.08 ^3,11^	[46]
White, healthy Danish men, aged 18–23 years, with no family history of diabetes		34 Participants without diabetes	0.57 ^3,12^	[43]
Participants with and without diabetes from 8 different studies		61 Participants without diabetes	Total gAUC: 0.34 ^1,11^	[42]
			Incremental gAUC: 0.40 ^1,11^	
Participants of European and African American descent, cases with a first-degree relative with T2DM, and normoglycemic controls with no family history of T2DM		336 Participants without diabetes and of Europid descent	0.16 ^1,9^	[58]
		157 Participants without diabetes and of African American decent	n. s. ^1,9^	

BMI, body mass index; gAUC, glucose area under the curve; n. s., not significant; OGTT, oral glucose tolerance test; rs, reference SNP; SNP, single nucleotide polymorphism; T2DM, type 2 diabetes mellitus; *TCF7L2*, *Transcription Factor 7 Like 2*; TÜF, Tübingen Family Study; *^,#,$^ SNPs within this gene locus are in high linkage disequilibrium (r^2^ > 0.8); ^+^ *p*-value as indicated in the report; ^1^ additive genetic model; ^2^ recessive genetic model; ^3^ dominant genetic model; ^4^ gAUC between homozygous carriers of the minor allele and the wild-type were compared; ^5^ gAUC between homozygous and heterozygous carriers of the minor allele were compared; ^6^ gAUC between heterozygous carriers of the minor allele and the wild-type were compared; ^7^ adjusted for age, sex; ^8^ adjusted for age, sex, family structure; ^9^ adjusted for age, sex, BMI; ^10^ adjusted for age, BMI; ^11^ no information about adjustment; ^12^ adjusted for birth weight; ^13^ non-independency of family members was statistically taken into account; ^14^ non-independency of twin pairs was statistically taken into account.

**Table 2:** Association between *peroxisome proliferator-activated receptor gamma* (*PPARγ*) SNP rs1801282 and gAUC after an OGTT in adults.

Study Characteristics	Sample Size Used	Results (*p*-Value) *	Reference
**EARS II** European men, aged 18–28 years, cases with a family history of premature acute myocardial infarction before the age of 55 years, and controls with a close birth date to the case	656 Participants without diabetes	0.99 ^1,5^	[22]
Japanese men with untreated essential hypertension	81 Participants without diabetes	n. s. ^4,6^	[38]
Unrelated, healthy white residents without diabetes, BMI < 40 kg/m^2^, living in Sicily	338 Participants without diabetes	n. s. ^2,7^	[37]
**Sapphire study** Family study with at least one sibling with hypertension, aged 35–60 years, Chinese or Japanese descent	1713 Participants without diabetes and hypertension	0.0210 ^2,8,13^ Significant differences between carriers of the minor allele (G) and the wild-type	[24]
Population-based study, elderly same-sex Danish twins, Caucasian descent	549 Participants without diabetes or treatment-naive participants	0.016 ^2,6,14^ Significantly lower gAUC in carriers of the minor allele (G) compared to the wild-type	[39]
	54 Dizygotic twin pairs without diabetes or treatment-naive participants	0.19 ^2,6,14^	
**Quebec Family Study** French-Canadian families (phase 1: randomly selected; phase 2/3: at least one person with obesity per family) living in and around the Quebec city area	663 Participants without diabetes	AUC_0–30_: 0.56 ^2,9^	[14]
		AUC_0–180_: 0.722 ^2,9^	
	680 Participants without diabetes or treatment-naive participants	0.52 ^3,10,13^	[15]
Healthy adults, aged 50–75 years, sedentary lifestyle, non-smoking, BMI < 37 kg/m^2^	32 Men without diabetes	n. s. ^4,11^	[40]
	41 Women without diabetes	n. s. ^4,11^	
**MICK** European men, aged 45–65 years, residency near Kiel	708 Participants without diabetes	0.48 ^1,11^	[25]
		0.386 ^3,6^	
	555 Participants with BMI < 30 kg/m^2^ and without diabetes	0.43 ^1,6^	
		0.382 ^3,6^	
Family study with at least 2 siblings with diagnosed T2DM before age 65 years, from Europe	144 Participants without diabetes	0.051 ^4,12^	[59]

BMI, body mass index; EARS II, European Atherosclerosis Research Study; gAUC, glucose area under the curve; MICK, Metabolic Intervention Cohort Kiel; n. s., not significant; OGTT, oral glucose tolerance test; *PPARγ, peroxisome proliferator-activated receptor gamma*; rs, reference SNP; SNP, single nucleotide polymorphism; T2DM, type 2 diabetes mellitus; * *p*-value as indicated in the report; ^1^ additive genetic model; ^2^ dominant genetic model; ^3^ recessive genetic model; ^4^ gAUC between heterozygous carriers of the minor allele and the wild-type were compared; ^5^ adjusted for age, center, case/control status; ^6^ no further information about adjustment; ^7^ adjusted for age, gender; ^8^ adjustment for age, sex, BMI, ethnicity; ^9^ adjusted for age, gender, BMI; ^10^ adjusted for age, sex, BMI; ^11^ adjusted for BMI; ^12^ adjusted for age, gender, BMI, systolic blood pressure, diastolic blood pressure, total cholesterol, triglycerides; ^13^ non-independency of family members was statistically taken into account; ^14^ non-independency of twin pairs was statistically taken into account.

**Table 3:** Associations between *potassium inwardly rectifying channel subfamily J member 11* (*KCNJ11*) SNPs and gAUC after an OGTT in adults.

Study Characteristics	SNP	Sample Size Used	Results (*p*-Value) ^+^	Reference
**Quebec Family Study** French-Canadian families (phase 1: randomly selected; phase 2/3: at least one person with obesity per family) living in and around the Quebec city area	rs1002227 *	669 Participants without diabetes or treatment-naive participants	n. s. ^1,5,11^	[13]
		712 Participants without diabetes or treatment-naive participants	n. s. ^1,5,11^	[12]
	rs11024273 *	669 Participants without diabetes or treatment-naive participants	n. s. ^1,5,11^	[13]
		712 Participants without diabetes or treatment-naive participants	n. s. ^1,5,11^	[12]
	rs2285676 *	669 Participants without diabetes or treatment-naive participants	n. s. ^1,5,11^	[13]
		712 Participants without diabetes or treatment-naive participants	n. s. ^1,5,11^	[12]
	rs5215 ^#^	669 Participants without diabetes or treatment-naive participants	n. s. ^1,5,11^	[13]
		712 Participants without diabetes or treatment-naive participants	n. s. ^1,5,11^	[12]
Han-Chinese participants with hypertension >140/90 mmHg or taking antihypertensive medication		667 Normoglycemic participants	n. s. ^1,6,12^	[35]
			n. s. ^2,6,12^	
			n. s. ^3,6^	
		458 Participants with impaired fasting glucose or impaired glucose tolerance	n. s. ^1,6^	
			n. s. ^2,6^	
			n. s. ^3,6^	
Unrelated participants without diabetes tested negative for GAD	rs5219 ^#^	298 Participants without diabetes	0.04 ^2,7^ Significantly higher gAUC in carriers of the minor allele (T) compared to the wild-type	[33]
			0.05 ^1,7^	
Unrelated participants without diabetes tested negative for GAD	rs5219 ^#^	298 Participants without diabetes	0.02 ^4,7^ Significantly higher gAUC in homozygous carriers of the minor allele (T) compared to the wild-type	[33]
		75 Participants without diabetes that underwent a hyperglycemic clamp	0.06 ^1,8^	
			0.17 ^4,8^	
		75 Participants without diabetes that underwent a hyperglycemic clamp	0.02 ^2,8^ Significantly higher gAUC in carriers of the minor allele (T) compared to the wild-type	
Healthy adults, aged 50–75 years, sedentary lifestyle, non-smoking, BMI < 37 kg/m^2^		214 Participants without diabetes	n. s. ^1,9^	[36]
Participants without diabetes, aged <65 years, in good health		461 Participants without diabetes	0.34 ^1,10^	[34]
Unrelated participants without diabetes, aged < 65 years, with no family history of diabetes		18 Participants without diabetes	n. s. ^4,10^	
Han-Chinese participants with hypertension >140/90 mmHg or taking antihypertensive medication		667 Normoglycemic participants	n. s. ^1,6^	[35]
			n. s. ^2,6^	
			n. s. ^3,6^	
		458 Participants with impaired fasting glucose or impaired glucose tolerance	n. s. ^1,6^	
			n. s. ^2,6^	
			n. s. ^3,6^	

BMI, body mass index; GAD, Generalized anxiety disorder; gAUC, glucose area under the curve; *KCNJ11*, *potassium inwardly rectifying channel subfamily J member 11*; n. s., not significant; OGTT, oral glucose tolerance test; rs, reference SNP; SNP, single nucleotide polymorphism; *^,#^ SNPs within this gene locus are in high linkage disequilibrium (r^2^ > 0.8); ^+^ *p*-value as indicated in the report; ^1^ additive genetic model; ^2^ dominant genetic model; ^3^ recessive genetic model; ^4^ gAUC between homozygous carriers of the minor allele and the wild-type were compared; ^5^ adjusted for age, sex; ^6^ adjusted for age, gender, BMI, mean systolic and diastolic blood pressure, angiotensin-converting enzyme inhibitor/angiotensin receptor blocker therapy; ^7^ adjusted for BMI, age, waist-to-hip ratio; ^8^ adjusted for BMI, age; ^9^ no further information about adjustment; ^10^ adjusted for age, race, BMI; ^11^ non-independency of family members was statistically taken into account; ^12^ Bonferroni-correction applied.

**Table 4:** Associations between SNPs and gAUC after an OGTT in adults. Gene loci are examined in at least three different articles.

Study Characteristics	SNP	Sample Size Used	Results (*p*-Value) ^+^	Reference
*ADIPOQ*				
**Quebec Family Study** French-Canadian families (phase 1: randomly selected; phase 2/3: at least one person with obesity per family) living in and around the Quebec city area	rs1501299	622 Participants without diabetes or treatment-naive participants	n.s ^1,6,23^	[16]
**Sapphire study** Family study with at least one sibling with hypertension, aged 35–60 years, Chinese or Japanese decent	rs2241766	1713 Participants without diabetes	n. s. ^2,7,23^	[24]
			n. s. ^1,7,23^	
**Quebec Family Study** French-Canadian families (phase 1: randomly selected; phase 2/3: at least one person with obesity per family) living in and around the Quebec city area		620 Participants without diabetes or treatment-naive participants	0.2 ^1,6,23^	[16]
**MICK** European men, aged 45–65 years, residency near Kiel	G11388A	110 Participants without diabetes or treatment-naive participants	n. s. ^2,8,24^	[26]
**Quebec Family Study** French-Canadian families (phase 1: randomly selected; phase 2/3: at least one person with obesity per family) living in and around the Quebec city area	rs822396	595 Participants without diabetes or treatment-naive participants	0.2 ^1,6,23^	[16]
*CDKAL1*				
**Quebec Family Study** French-Canadian families (phase 1: randomly selected; phase 2/3: at least one person with obesity per family) living in and around the Quebec city area	rs10946403	669 Participants without diabetes or treatment-naive participants	n. s. ^1,9,23^	[13]
		712 Participants without diabetes or treatment-naive participants	n. s. ^1,9,23^	[12]
	rs523069		n. s. ^1,10,23^	
**EUGENE2** European, non-diabetic offspring of one parent with T2DM and one parent without T2DM	rs7754840 *	846 Participants without diabetes	0.016 ^1,11^ Significant difference between homozygous and heterozygous carriers of the minor allele (C) and the wild-type	[28]
		698 Normoglycemic participants	0.005 ^1,11^ Significant difference between homozygous and heterozygous carriers of the minor allele (C) and the wild-type	
		148 Participants with impaired glucose tolerance and/or impaired fasting glucose	n. s. ^1,11^	
		100 Participants from Gothenburg without diabetes	0.233 ^1,11^	
**EUGENE2** European, non-diabetic offspring of one parent with T2DM and one parent without T2DM	rs7754840 *	100 Participants from Gothenburg without diabetes	0.233 ^1,11^	[28]
		110 Participants from Catanzaro without diabetes	0.242 ^1,11^	
		270 Participants from Copenhagen without diabetes	0.007 ^1,11^ Significant difference between homozygous and heterozygous carriers of the minor allele (C) and the wild-type	
		217 Participants from Kuopio without diabetes	0.346 ^1,11^	
		149 Participants from Tuebingen without diabetes	0.521 ^1,11^	
**METSIM** Finnish men, aged 45–73 years, randomly selected from the population register of Kuopio		2405 Normoglycemic participants	0.694 ^1,11^	
		3367 Participants without diabetes	n. s. ^1,8^	
**TÜF** German participants with an increased risk of diabetes		1065 Participants without diabetes	0.02 ^1,8^ Significant difference between homozygous and heterozygous carriers of the minor allele (C) and the wild-type	[21]
**Quebec Family Study** French-Canadian families (phase 1: randomly selected; phase 2/3: at least one person with obesity per family) living in and around the Quebec city area	rs7756992 *	669 Participants without diabetes or treatment-naive participants	n. s. ^1,9,23^	[13]
*CDKN2A/B*				
**Quebec Family Study** French-Canadian families (phase 1: randomly selected; phase 2/3: at least one person with obesity per family) living in and around the Quebec city area	rs10811661	669 Participants without diabetes or treatment-naive participants	n. s. ^1,9,23^	[13]
		712 Participants without diabetes or treatment-naive participants	<0.05 ^1,10,23^ Significant difference between homozygous and heterozygous carriers of the minor allele (C) and the wild-type	[12]
**TÜF** German participants with an increased risk of diabetes		1065 Participants without diabetes	0.09 ^1,8^	[21]
	rs3217992	669 Participants without diabetes or treatment-naive participants	n. s. ^1,9,23^	[13]
	rs3731201		n. s. ^1,9,23^	
		712 Participants without diabetes or treatment-naive participants	n. s. ^1,9,23^	[12]
	rs3731211	669 Participants without diabetes or treatment-naive participants	n. s. ^1,9,23^	[13]
**Quebec Family Study** French-Canadian families (phase 1: randomly selected; phase 2/3: at least one person with obesity per family) living in and around the Quebec city area	rs3731211	712 Participants without diabetes or treatment-naive participants	n. s. ^1,9,23^	[12]
	rs495490	669 Participants without diabetes or treatment-naive participants	n. s. ^1,9,23^	[13]
		712 Participants without diabetes or treatment-naive participants	n. s. ^2,9,23^	[12]
	rs523096 *	669 Participants without diabetes or treatment-naive participants	n. s. ^1,9,23^	[13]
		721 Participants without diabetes or treatment-naive participants	n. s. ^1,10,23^	[12]
	rs564398 *	669 Participants without diabetes or treatment-naive participants	n. s. ^1,9,23^	[13]
		712 Participants without diabetes or treatment-naive participants	n. s. ^1,9,23^	[12]
*HHEX*				
**MESYBEPO** German case–control study, participants with or without metabolic syndrome and healthy or disturbed glucose metabolism, living in Berlin or Potsdam area	rs1111875 *	681 Participants without diabetes	0.45 ^1,12^	[29]
		411 Normoglycemic participants	0.18 ^1,12^	
**EUGENE2** European, non-diabetic offspring of one parent with T2DM and one parent without T2DM		844 Participants without diabetes	0.9 ^1,13^	[27]
**Quebec Family Study** French-Canadian families (phase 1: randomly selected; phase 2/3: at least one person with obesity per family) living in and around the Quebec city area		669 Participants without diabetes or treatment-naive participants	n. s. ^1,9,23^	[13]
		712 Participants without diabetes or treatment-naive participants	n. s. ^1,9,23^	[12]
**TÜF** German participants with an increased risk of diabetes		1065 Participants without diabetes	0.04 ^1,8^ Significantly higher gAUC in homozygous carriers of the minor allele (T) compared to the wild-type	[21]
**Quebec Family Study** French-Canadian families (phase 1: randomly selected; phase 2/3: at least one person with obesity per family) living in and around the Quebec city area	rs7911264	669 Participants without diabetes or treatment-naive participants	n. s. ^1,9,23^	[13]
**MESYBEPO** German case–control study, participants with or without metabolic syndrome and healthy or disturbed glucose metabolism, living in Berlin or Potsdam area	rs7923837 *	680 Participants without diabetes	0.29 ^1,12^	[29]
		410 Normoglycemic participants	0.58 ^1,12^	
**EUGENE2** European, non-diabetic offspring of one parent with T2DM and one parent without T2DM		842 Participants without diabetes	0.9 ^1,13^	[27]
**Quebec Family Study** French-Canadian families (phase 1: randomly selected; phase 2/3: at least one person with obesity per family) living in and around the Quebec city area		669 Participants without diabetes or treatment-naive participants	n. s. ^1,9,23^	[13]
		712 Participants without diabetes or treatment-naive participants	n. s. ^1,9,23^	[12]
**TÜF** German participants with an increased risk of diabetes		1065 Participants without diabetes	0.05 ^1,8^	[21]
*HNF4α*				
**Amish Family Diabetes Study** Old Order Amish population, participants with previously diagnosed T2DM and first- and second-degree relatives, aged ≥18 years	rs1884614	698 Participants without diabetes or treatment-naive participants	0.022 ^1,9,23^ Significant difference between homozygous and heterozygous carriers of the minor allele (T) and the wild-type	[19]
			0.01 ^2,9,23^ Significantly higher gAUC in carriers of the minor allele (T) compared to the wild-type	
**Inter99** Population-based study, participants aged 30–60 years, Caucasian descent		4430 Normoglycemic participants	0.05 ^1,10^	[31]
			0.21 ^3,10^	
			0.02 ^2,10^ Significant difference between carriers of the minor allele (A) and the wild-type	
	rs1885088	4336 Normoglycemic participants	n. s. ^1,10^	
			n. s. ^3,10^	
			n. s. ^2,10^	
**Quebec Family Study** French-Canadian families (phase 1: randomly selected; phase 2/3: at least one person with obesity per family) living in and around the Quebec city area		524 Participants without diabetes	0.06 ^1,14^	[17]
		n. a. Participants without diabetes and a high physical activity level	0.01 ^4,14^ Significant difference between homozygous and heterozygous carriers of the minor allele (A)	
			0.01 ^5,14^ Significant difference between homozygous carriers of the minor allele (A) and the wild-type	
		n. a. Participants without diabetes and a low physical activity level	n. s. ^4,14^	[17]
			n. s. ^5,14^	
**Inter99** Population-based study, participants aged 30–60 years, Caucasian decent	rs2425637	4394 Normoglycemic participants	n. s. ^1,10^	[31]
			n. s. ^3,10^	
			n. s. ^2,10^	
**Amish Family Diabetes Study** Old Order Amish population, participants with previously diagnosed T2DM and first- and second-degree relatives, aged ≥ 18 years	rs2425640	698 Participants without diabetes or treatment-naive participants	n. s. ^1,9,23^	[19]
			n. s. ^2,9,23^	
**Inter99** Population-based study, participants aged 30–60 years, Caucasian decent	rs3818247	4413 Normoglycemic participants	n. s. ^1,10^	[31]
			n. s. ^3,10^	
			n. s. ^2,10^	
**Quebec Family Study** French-Canadian families (phase 1: randomly selected; phase 2/3: at least one person with obesity per family) living in and around the Quebec city area	rs745975	505 Participants without diabetes	0.17 ^1,14^	[17]
*IGF2BP2*				
**Quebec Family Study** French-Canadian families (phase 1: randomly selected; phase 2/3: at least one person with obesity per family) living in and around the Quebec city area	rs4402960	669 Participants without diabetes or treatment-naive participants	n. s. ^1,9,23^	[13]
		712 Participants without diabetes or treatment-naive participants	<0.05 ^1,10,23^ Significant difference between carriers of the minor allele (T) and the wild-type	[12]
**TÜF** German participants with an increased risk of diabetes		1065 Participants without diabetes	0.34 ^1,8^	[21]
*IL-6*				
Healthy, non-smoking French-Canadian men, living in the Quebec area	rs1800795 *	270 Participants without diabetes	0.43 ^2,15^	[51]
			0.42 ^2,16^	
Healthy Caucasians with BMI < 40 kg/m^2^		32 Participants without diabetes	0.001 ^3,8^ Significantly lower gAUC in carriers of the minor allele (C) compared to the wild allele	[52]
**Inter99** Population-based study, participants aged 30–60 years, Caucasian descent		4401 Normoglycemic participants	0.51 ^1,17^	[30]
			0.25 ^3,17^	
			0.58 ^2,17^	
Healthy participants, aged 50–75 years, sedentary lifestyle, non-smoking, BMI < 37 kg/m^2^		87 Participants without diabetes	n. s. ^2,18^	[55]
**Inter99** Population-based study, participants aged 30–60 years, Caucasian descent	rs1800797 *	4401 Normoglycemic participants	0.48 ^1,17^	[30]
			0.7 ^3,17^	
			0.3 ^2,17^	
*PC-1*				
Asian Indians and Caucasians without diabetes	rs1044498	158 Asian Indian participants without diabetes or treatment-naive participants	n. s. ^2,8^	[49]
		152 Caucasian participants without diabetes or treatment-naive participants	n. s. ^2,8^	
Unrelated, healthy, volunteers without diabetes, aged 20–59 years, BMI < 30.0 kg/m^2^		118 Normoglycemic participants	n. s. ^1,17,24^	[54]
Unrelated Caucasians without diabetes living in Sicily		211 Participants without diabetes, but with obesity	n. s. ^2,20,24^	[53]
		220 Participants without diabetes and obesity	>0.05 ^2,20,24^	
		431 Participants without diabetes	n. s. ^2,20,24^	
Unrelated, healthy white residents without diabetes, BMI < 40 kg/m^2^, living in Sicily		338 Participants without diabetes	n. s. ^2,19^	[37]
		764 Participants without diabetes	0.05 ^5,10^	[50]
		764 Participants without diabetes	0.02 ^1,10^ Significant difference between homozygous and heterozygous carriers of the minor allele (C) and the wild-type	
		475 Participants without diabetes but with obesity	0.048 ^1,10^ Significant difference between homozygous and heterozygous carriers of the minor allele (C) and the wild-type	
		289 Participants without diabetes and obesity	n. s. ^1,10^	
*SLC30A8*				
**Quebec Family Study** French-Canadian families (phase 1: randomly selected; phase 2/3: at least one person with obesity per family) living in and around the Quebec city area.	rs13266634	669 Participants without diabetes	n. s. ^1,9,23^	[13]
		712 Participants without diabetes	n. s. ^1,9,23^	[12]
**TÜF** German participants with an increased risk of diabetes		1065 Participants without diabetes or treatment-naive participants	0.27 ^1,8^	[21]
*TNF-α*				
40 families with obesity but without diabetes, genetic trait of obesity, Caucasian origin	rs1800629	122 Participants without diabetes	0.077 ^2,8^	[57]
		38 Men without diabetes	0.105 ^2,8^	
		83 Women without diabetes	0.298 ^2,8^	
**EARS II** European men, aged 18–28 years, cases with a family history of premature acute myocardial infarction before the age of 55 years, and controls with a close birth date to the case		335 Cases without diabetes	0.57 ^2,21^	[23]
		323 Controls without diabetes	0.85 ^2,21^	
Hypertensive participants without diabetes and unrelated, healthy, non-diabetic, normotensive participants with first-degree relatives free of diabetes, Asian descent		177 Participants without diabetes, but with hypertension	0.750 ^2,22^	[56]
		202 Normotensive participants without diabetes	0.132 ^2,22^	
**BErG-Study** Unrelated German Caucasian population without diabetes, wide range of BMI, with and without hypertension or impaired glucose tolerance		176 Participants without diabetes	n. s. ^2,10^	[32]

*ADIPOQ*, *adiponectin*; BErG-Study, Berlin Ernährung Geschwister Study; BMI, body mass index; *CDKAL1*, *CDK5 regulatory subunit-associated protein 1 like 1*; *CDKN2A/B*, *cyclin-dependent kinase inhibitor 2A/B*; EARS II, European Atherosclerosis Research Study; EUGENE2, European network on Functional Genomics of Type 2 Diabetes; gAUC, glucose area under the curve; *HHEX*, *hematopoietically expressed homeobox*; *HNF4α*, *hepatocyte nuclear factor 4 alpha*; HOMA-IR, homeostasis model assessment; *IGF2BP2*, *insulin-like growth factor 2 mRNA-binding protein 2*; Inter99, Lifestyle Intervention in a General Population for Prevention of Ischaemic Heart Disease; *IL-6*, *interleukin 6*; MESYBEPO, Metabolic Syndrome Berlin Potsdam study; METSIM, Metabolic Syndrome in Men; MICK, Metabolic Intervention Cohort Kiel; n. a., not available; n. s., not significant; OGTT, oral glucose tolerance test; *PC-1*, *proprotein convertase 1*; *SLC30A8, solute carrier family 30 member 8*; rs, reference SNP; SNP, single nucleotide polymorphism; T2DM, type 2 diabetes mellitus; *TNF-α*, *tumor necrosis factor α*; TÜF, Tübinger Family Study; * SNPs within this gene locus are in high linkage disequilibrium (r^2^ > 0.8); ^+^ *p*-value as indicated in the report; ^1^ additives genetic model; ^2^ dominate genetic model; ^3^ recessive genetic model; ^4^ gAUC between homozygous and heterozygous carriers of the minor allele were compared; ^5^ gAUC between homozygous carriers of the minor allele and the wild-type were compared; ^6^ adjusted for age, age^2^, sex, fat mass; ^7^ adjusted for age, sex, BMI, ethnicity, area of enrolment; ^8^ no further information about adjustment; ^9^ adjusted for age, sex; ^10^ adjusted for age, sex, BMI; ^11^ adjusted for age, BMI, HOMA-IR, gender, family center; ^12^ adjusted for age, gender, BMI, waist circumference; ^13^ adjusted for center, family relationship, sex, age, BMI; ^14^ adjusted for age, age^2^, sex, BMI; ^15^ adjusted for age, waist circumference; ^16^ adjusted for age, BMI; ^17^ adjusted for age, gender, BMI; ^18^ adjusted for age, gender, ethnicity; ^19^ adjusted for age, gender; ^20^ adjusted for age, gender, waist circumference; ^21^ adjusted for age, center, BMI, waist/hip ratio, smoking status, physical activity, fasting glucose; ^22^ adjusted for age, BMI, body fat; ^23^ non-independency of family members was statistically taken into account; ^24^ Bonferroni-correction applied.

## Data Availability

The data that support the findings of this study are available from the corresponding author upon reasonable request.

## Supplementary Materials

The following supporting information can be downloaded at: https://www.mdpi.com/article/10.3390/nu15071695/s1, Table S1: Identified genes for an association between SNPs and gAUC after an OGTT in adults; Table S2: Associations between SNPs and gAUC after an OGTT in adults. References [80,81,82,83,84,85,86,87,88,89,90,91,92,93,94,95,96,97,98,99,100,101,102,103,104,105,106,107,108,109,110,111,112,113,114,115,116,117,118,119,120,121,122,123,124,125,126,127,128,129,130,131,132,133,134,135,136,137,138,139,140,141,142,143,144,145,146,147,148,149,150,151,152,153,154,155,156,157,158,159,160,161,162,163,164,165,166,167] are cited in the Supplementary Materials.
