# Supplementary material for: Association between Genotype and the Glycemic Response to an Oral Glucose Tolerance Test: A Systematic Review
Source: Nutrients. 2023 Mar 30;15(7):1695. doi: 10.3390/nu15071695 (PMC10096950; doi:10.3390/nu15071695)
Supplement: Supplementary file 1 [file nutrients-15-01695-s001.zip › Table S1.pdf]

Contact Info: Christina Holzapfel, PhD, Institute for Nutritional Medicine, School of Medicine, Technical University of Munich, Georg-Brauchle-Ring 62, 80992 Munich. E-Mail: christina.holzapfel@tum.de; Phone: 0049 89 289 249 23; Fax: 0049 89 289 249 22

Supplementary Table S1. Identified genes for an association between SNPs and gAUC after an OGTT in adults. Gene loci are examined in one article.

Gene	Name	Author *et al.*	Year	Reference
ACADM	Acyl-CoA Dehydrogenase Medium Chain	Hornbak *et al.*	2011	[1]
ACADS	Acyl-CoA Dehydrogenase Short Chain	Hornbak *et al.*	2011	[1]
ACE	Angiotensin I Converting Enzyme	Ohishi *et al.*	2000	[2]
ADCY5	Adenylate Cyclase 5	Vasan *et al.*	2011	[3]
AdipoR1	Adiponectin Receptor 1	Ruchat *et al.*	2008a	[4]
AdipoR2	Adiponectin Receptor 2	Ruchat *et al.*	2008a	[4]
AHSG	Alpha 2-HS Glycoprotein	Andersen *et al.*	2008	[5]
AKT1	AKT Serine/Threonine Kinase 1	McKenzie *et al.*	2011	[6]
AMPKγ2	5´-AMP-Activated Protein Kinase γ2 Gene	Xu *et al.*	2005	[7]
ANGPTL4	Angiopoietin Like 4	Staiger *et al.*	2008	[8]
APOA4/5	Apolipoprotein A4/5	Martin *et al.*	2003	[9]
APOE	Apolipoprotein E	Ragogna *et al.*	2012	[10]
ARHGEF11	Rho Guanine Nucleotide Exchange Factor 11	Fu *et al.*	2007	[11]
BCHE	Butyrylcholinesterase	Johansen *et al.*	2004	[12]
BTC	Betacellulin	Silver *et al.*	2007	[13]
CASQ1	Calsequestrin 1	Fu *et al.*	2004	[14]
CCNL1	Cyclin L1	Vasan *et al.*	2011	[3]
CD36	CD36 Molecule	Jayewardene *et al.*	2016	[15]
CPT1b	Carnitine Palmitoyltransferase 1B	Auinger *et al.*	2013	[16]
CPT2	Carnitine Palmitoyltransferase 2	Auinger *et al.*	2013	[16]
CTF1	Cardiotrophin 1	Lutz *et al.*	2014	[17]
CTGF	Connective Tissue Growth Factor	Pivovarova *et al.*	2011	[18]
FATP6	Fatty Acid Transport Protein 6	Auinger *et al.*	2012	[19]
FOXA2	Forkhead Box A2	Banasik *et al.*	2012	[20]
FOXO1	Forkhead Box O1	Mussig *et al.*	2009a	[21]
FOXO3A	Forkhead Box O3A	Banasik *et al.*	2011	[22]
G6PC2/ABCB11	Glucose-6-Phosphatase Catalytic Subunit 2/<br>ATP Binding Cassette Subfamily B Member 11	Rose *et al.*	2009	[23]
GCKR	Glucokinase Regulator	Sparso *et al.*	2008	[24]
GNB3	G Protein Subunit Beta 3	Nurnberger *et al.*	2003	[25]
GPBAR1	G Protein-Coupled Bile Acid Receptor 1	Mussig *et al.*	2009b	[26]
GPR40	Fatty Acid Transport Protein 6	Hamid *et al.*	2005	[27]
Ghrelin	Ghrelin	Poykko *et al.*	2003	[28]
HK1	Hexokinase 1	Gjesing *et al.*	2011	[29]
IL-1B	Interleukin 1 Beta	Luotola *et al.*	2009	[30]
IL6R	Interleukin 6 Receptor	Hamid *et al.*	2004	[31]
KCNH2	Potassium Voltage-Gated Channel Subfamily H Member 2	Engelbrechtsen *et al.*	2018	[32]
KCNQ1	Potassium Voltage-Gated Channel Subfamily Q Member 1	Holmkvist *et al.*	2009	[33]
KLF11	Kruppel Like Factor 11	Neve *et al.*	2005	[34]
LEPR	Leptin Receptor	Wauters *et al.*	2001	[35]

Gene	Name	Author *et al.*	Year	Reference
LGR4	Leucine Rich Repeat Containing G Protein-Coupled Receptor 4	Zou *et al.*	2017	[36]
LPIN1	Lipin 1	Burgdorf *et al.*	2010	[37]
LPIN2	Lipin 2	Aulchenko *et al.*	2007	[38]
LPL	Lipoprotein Lipase	Ukkola *et al.*	2001	[39]
MAP4K5	Mitogen-Activated Protein Kinase Kinase Kinase Kinase 5	Gu *et al.*	2006	[40]
MG53	Skeletal-Muscle-Specific E3 Ubiquitin Ligase	Yang *et al.*	2016	[41]
MTMR9	Myotubularin Related Protein 9	Tang *et al.*	2014	[42]
OPRM1	Opioid Receptor Mu 1	Ruchat *et al.*	2008b	[43]
PCSK1	Proprotein Convertase Subtilisin/Kexin Type 1	Heni *et al.*	2010	[44]
PCSK2	Proprotein Convertase Subtilisin/Kexin Type 2	Chang *et al.*	2015	[45]
PGC-1a	Peroxisome Proliferator–Activated Receptor Coactivator-1α	Oberkofler *et al.*	2004	[46]
PIK3CG	Phosphatidylinositol-4,5-Bisphosphate 3-Kinase Catalytic Subunit Gamma	Kachele *et al.*	2015	[47]
PLA2G6	Phospholipase A2 Group VI	Yan *et al.*	2015	[48]
PPARα	Peroxisome Proliferator-Activated Receptor Alpha	Bosse *et al.*	2003	[49]
PPARγC1A	PPARG Coactivator 1 Alpha	Ruchat *et al.*	2009c	[50]
PTBP1	Polypyrimidine Tract Binding Protein 1	Hansen *et al.*	2015	[51]
PTGES2	Prostaglandin E Synthase 2	Lindner *et al.*	2007	[52]
PYY	Peptide YY	Torekov *et al.*	2005	[53]
QPCTL	Glutaminyl-Peptide Cyclotransferase Like	Burgdorf *et al.*	2012	[54]
RETN	Resistin	Bouchard *et al.*	2004	[55]
SGLT2	Sodium–Glucose Cotransporter 2	Enigk *et al.*	2011	[56]
SGNE1	Secretory Granule Neuroendocrine Protein 1	Bouatia-Naji *et al.*	2007	[57]
SOCS7	Suppressor Of Cytokine Signaling 7	Capuano *et al.*	2013	[58]
SUR1	Sulfonylurea Receptor 1	Weisnagel *et al.*	2001	[59]
TRPM5	Transient Receptor Potential Cation Channel Subfamily M Member 5	Ketterer *et al.*	2011	[60]
UCP3	Uncoupling Protein 3	Halsall *et al.*	2001	[61]
USF1	Upstream Transcription Factor 1	Putt *et al.*	2004	[62]
Vasfatin gene	Vasfatin Gene	Jian *et al.*	2006	[63]
VDR	Vitamin D Receptor	Chiu *et al.*	2001	[64]

gAUC, glucose area under the curve; OGTT, oral glucose tolerance test; SNP, single nucleotide polymorphism

References

1. Hornbak M, Banasik K, Justesen JM, Krarup NT, Sandholt CH, Andersson A, Sandbaek A, Lauritzen T, Pisinger C, Witte DR, et al. The minor C-allele of rs2014355 in ACADS is associated with reduced insulin release following an oral glucose load. Bmc Medical Genetics. 2011;12:8.
2. Ohishi M, Rakugi H, Miki T, Katsuya T, Okamura A, Kamide K, Nakata Y, Takami S, Ikegami H, Yanagitani Y, et al. Deletion polymorphism of angiotensin-converting enzyme gene is associated with postprandial hyperglycaemia in individuals undergoing general check-up. Clin Exp Pharmacol Physiol. 2000;27(7):483-7.
3. Vasan SK, Neville MJ, Antonisamy B, Samuel P, Fall CH, Geethanjali FS, Thomas N, Raghupathy P, Brismar K, Karpe F. Absence of Birth-Weight Lowering Effect of ADCY5 and Near CCNL, but Association of Impaired Glucose-Insulin Homeostasis with ADCY5 in Asian Indians. PLoS One. 2011;6(6):6.
4. Ruchat SM, Loos RJF, Rankinen T, Vohl MC, Weisnagel SJ, Despres JP, Bouchard C, Perusse L. Associations between glucose tolerance, insulin sensitivity and insulin secretion phenotypes and polymorphisms in adiponectin and adiponectin receptor genes in the Quebec Family Study. Diabetic Medicine. 2008;25(4):400-6.
5. Andersen G, Burgdorf KS, Sparso T, Borch-Johnsen K, Jorgensen T, Hansen T, Pedersen O. AHSG tag single nucleotide Polymorphisms associate with type 2 diabetes and dyslipidemia: Studies of metabolic traits in 7,683 white danish subjects. Diabetes. 2008;57(5):1427-32.

6. McKenzie JA, Witkowski S, Ludlow AT, Roth SM, Hagberg JM. AKT1 G205T genotype influences obesity-related metabolic phenotypes and their responses to aerobic exercise training in older Caucasians. Exp Physiol. 2011;96(3):338-47.
7. Xu M, Li XY, Wang JG, Du PF, Hong J, Gu WQ, Zhang YF, Ning G. Glucose and lipid metabolism in relation to novel polymorphisms in the 5 '-AMP-activated protein kinase gamma 2 gene in Chinese. Mol Genet Metab. 2005;86(3):372-8.
8. Staiger H, Machicao F, Werner R, Guirguis A, Weisser M, Stefan N, Fritsche A, Haring HU. Genetic variation within the ANGPTL4 gene is not associated with metabolic traits in white subjects at an increased risk for type 2 diabetes mellitus. Metab-Clin Exp. 2008;57(5):637-43.
9. Martin S, Nicaud V, Humphries SE, Talmud PJ, Group E. Contribution of APOA5 gene variants to plasma triglyceride determination and to the response to both fat and glucose tolerance challenges. Biochim Biophys Acta-Mol Basis Dis. 2003;1637(3):217-25.
10. Ragogna F, Lattuada G, Ruotolo G, Luzi L, Perseghin G. Lack of association of apoE epsilon 4 allele with insulin resistance. Acta Diabetol. 2012;49(1):25-32.
11. Fu M, Sabra MM, Damcott C, Pollin TI, Ma LJ, Ott S, Shelton JC, Shi XL, Reinhart L, O'Connell J, et al. Evidence that Rho guanine nucleotide exchange factor 11 (ARHGEF11) on 1q21 is a type 2 diabetes susceptibility gene in the old order Amish. Diabetes. 2007;56(5):1363-8.
12. Johansen A, Nielsen EMD, Andersen G, Hamid YH, Jensen DP, Glumer C, Drivsholm T, Borch-Johnsen K, Jorgensen T, Hansen T, et al. Large-scale studies of the functional K variant of the butyrylcholinesterase gene in relation to Type 2 diabetes and insulin secretion. Diabetologia. 2004;47(8):1437-41.
13. Silver KD, Shi X, Mitchell BD. Betacellulin variants and type 2 diabetes in the Old Order Amish. Exp Clin Endocrinol Diabetes. 2007;115(4):229-31.
14. Fu M, Damcott CM, Sabra M, Pollin TI, Ott SH, Wang J, Garant MJ, O'Connell JR, Mitchell BD, Shuldiner AR. Polymorphism in the calsequestrin 1 (CASQ1) gene on chromosome 1q21 is associated with type 2 diabetes in the old order Amish. Diabetes. 2004;53(12):3292-9.
15. Jayewardene AF, Mavros Y, Gwinn T, Hancock DP, Rooney KB. Associations between CD36 gene polymorphisms and metabolic response to a short-term endurance-training program in a young-adult population. Appl Physiol Nutr Metab. 2016;41(2):157-67.
16. Auinger A, Rubin D, Sabandal M, Helwig U, Ruther A, Schreiber S, Foelsch UR, Doring F, Schrezenmeir J. A common haplotype of carnitine palmitoyltransferase 1b is associated with the metabolic syndrome. Br J Nutr. 2013;109(5):810-5.
17. Lutz SZ, Franck O, Bohm A, Machann J, Schick F, Machicao F, Fritsche A, Haring HU, Staiger H. Common Genetic Variation in the Human CTF1 Locus, Encoding Cardiotrophin-1, Determines Insulin Sensitivity. PLoS One. 2014;9(7):8.
18. Pivovarova O, Fisher E, Dudziak K, Ilkavets I, Dooley S, Slominsky P, Limborska S, Weickert MO, Spranger J, Fritsche A, et al. A polymorphism within the connective tissue growth factor (CTGF) gene has no effect on non-invasive markers of beta-cell area and risk of type 2 diabetes. Dis Markers. 2011;31(4):241-6.
19. Auinger A, Helwig U, Pfeuffer M, Rubin D, Luedde M, Rausche T, El Mokhtari NE, Folsch UR, Schreiber S, Frey N, et al. A variant in the heart-specific fatty acid transport protein 6 is associated with lower fasting and postprandial TAG, blood pressure and left ventricular hypertrophy. Br J Nutr. 2012;107(10):1422-8.
20. Banasik K, Hollensted M, Andersson E, Sparso T, Sandbaek A, Lauritzen T, Jorgensen T, Witte DR, Pedersen O, Hansen T. The effect of FOXA2 rs1209523 on glucose-related phenotypes and risk of type 2 diabetes in Danish individuals. Bmc Medical Genetics. 2012;13:7.
21. Mussig K, Staiger H, Machicao F, Stancakova A, Kuusisto J, Laakso M, Thamer C, Machann J, Schick F, Claussen CD, et al. Association of Common Genetic Variation in the FOXO1 Gene with beta-Cell Dysfunction, Impaired Glucose Tolerance, and Type 2 Diabetes. J Clin Endocrinol Metab. 2009;94(4):1353-60.
22. Banasik K, Ribel-Madsen R, Gjesing AP, Wegner L, Andersson A, Poulsen P, Borglykke A, Witte DR, Pedersen O, Hansen T, et al. The FOXO3A rs2802292 G-allele associates with improved peripheral and hepatic insulin sensitivity and increased skeletal muscle-FOXO3A mRNA expression in twins. J Clin Endocrinol Metab. 2011;96(1):E119-24.
23. Rose CS, Grarup N, Krarup NT, Poulsen P, Wegner L, Nielsen T, Banasik K, Faerch K, Andersen G, Albrechtsen A, et al. A variant in the G6PC2/ABCB11 locus is associated with increased fasting plasma glucose, increased basal hepatic glucose production and increased insulin release after oral and intravenous glucose loads. Diabetologia. 2009;52(10):2122-9.
24. Sparso T, Andersen G, Nielsen T, Burgdorf KS, Gjesing AP, Nielsen AL, Albrechtsen A, Rasmussen SS, Jorgensen T, Borch-Johnsen K, et al. The GCKR rs780094 polymorphism is associated with elevated fasting serum triacylglycerol, reduced fasting and OGTT-related insulinaemia, and reduced risk of type 2 diabetes. Diabetologia. 2008;51(1):70-5.
25. Nurnberger J, Dammer S, Philipp T, Wenzel RR, Schafers RF. Metabolic and haemodynamic effects of oral glucose loading in young healthy men carrying the 825T-allele of the G protein beta 3 subunit. Cardiovasc Diabetol. 2003;2:7.
26. Mussig K, Staiger H, Machicao F, Machann J, Schick F, Schafer SA, Claussen CD, Holst JJ, Gallwitz B, Stefan N, et al. Preliminary report: genetic variation within the GPBAR1 gene is not associated with metabolic traits in white subjects at an increased risk for type 2 diabetes mellitus. Metab-Clin Exp. 2009;58(12):1809-11.
27. Hamid YH, Vissing H, Holst B, Urhammer SA, Pyke C, Hansen SK, Glumer C, Borch-Johnsen K, Jorgensen T, Schwartz TW, et al. Studies of relationships between variation of the human G protein-coupled receptor 40 Gene and Type 2 diabetes and insulin release. Diabetic Medicine. 2005;22(1):74-80.
28. Poykko S, Ukkola O, Kauma H, Savolainen MJ, Kesaniemi YA. Ghrelin Arg51Gln mutation is a risk factor for Type 2 diabetes and hypertension in a random sample of middle-aged subjects. Diabetologia. 2003;46(4):455-8.

29. Gjesing AP, Nielsen AA, Brandslund I, Christensen C, Sandbaek A, Jorgensen T, Witte D, Bonnefond A, Froguel P, Hansen T, et al. Studies of a genetic variant in HK1 in relation to quantitative metabolic traits and to the prevalence of type 2 diabetes. Bmc Medical Genetics. 2011;12:8.
30. Luotola K, Paakkonen R, Alanne M, Lanki T, Moilanen L, Surakka I, Pietila A, Kahonen M, Nieminen MS, Kesaniemi YA, et al. Association of Variation in the Interleukin-1 Gene Family with Diabetes and Glucose Homeostasis. J Clin Endocrinol Metab. 2009;94(11):4575-83.
31. Hamid YH, Urhammer SA, Jensen DP, Glumer C, Borch-Johnsen K, Jorgensen T, Hansen T, Pedersen O. Variation in the interleukin-6 receptor gene associates with type 2 diabetes in Danish whites. Diabetes. 2004;53(12):3342-5.
32. Engelbrechtsen L, Mahendran Y, Jonsson A, Gjesing AP, Weeke PE, Jorgensen ME, Faerch K, Witte DR, Holst JJ, Jorgensen T, et al. Common variants in the hERG (KCNH2) voltage-gated potassium channel are associated with altered fasting and glucose-stimulated plasma incretin and glucagon responses. BMC Genet. 2018;19:9.
33. Holmkvist J, Banasik K, Andersen G, Unoki H, Jensen TS, Pisinger C, Borch-Johnsen K, Sandbaek A, Lauritzen T, Brunak S, et al. The type 2 diabetes associated minor allele of rs2237895 KCNQ1 associates with reduced insulin release following an oral glucose load. PLoS One. 2009;4(6):e5872.
34. Neve B, Fernandez-Zapico ME, Ashkenazi-Katalan V, Dina C, Hamid YH, Joly E, Vaillant E, Benmezroua Y, Durand E, Bakaher N, et al. Role of transcription factor KLF11 and its diabetes-associated gene variants in pancreatic beta cell function. Proc Natl Acad Sci U S A. 2005;102(13):4807-12.
35. Wauters M, Mertens I, Rankinen T, Chagnon M, Bouchard C, Van Gaal L. Leptin receptor gene polymorphisms are associated with insulin in obese women with impaired glucose tolerance. J Clin Endocrinol Metab. 2001;86(7):3227-32.
36. Zou YY, Ning TL, Shi J, Chen MP, Ding L, Huang Y, Kauderer S, Xu M, Cui B, Bi YF, et al. Association of a Gain-of-Function Variant in LGR4 with Central Obesity. Obesity. 2017;25(1):252-60.
37. Burgdorf KS, Sandholt CH, Sparso T, Andersen G, Witte DR, Jorgensen T, Sandbaek A, Lauritzen T, Sorensen TIA, Madsbad S, et al. Studies of association between LPIN1 variants and common metabolic phenotypes among 17 538 Danes. Eur J Endocrinol. 2010;163(1):81-7.
38. Aulchenko YS, Pullen J, Kloosterman WP, Yazdanpanah M, Hofman A, Vaessen N, Snijders P, Zubakov D, Mackay I, Olavesen M, et al. LPIN2 is associated with type 2 diabetes, glucose metabolism, and body composition. Diabetes. 2007;56(12):3020-6.
39. Ukkola O, Garenc C, Perusse L, Bergeron J, Despres JP, Rao DC, Bouchard C. Genetic variation at the lipoprotein lipase locus and plasma lipoprotein and insulin levels in the Quebec Family Study. Atherosclerosis. 2001;158(1):199-206.
40. Gu YY, Luo TH, Yang J, Zhang D, Dai M, Jian WX, Zheng S. The-822G/A polymorphism in the promoter region of the MAP4K5 gene is associated with reduced risk of type 2 diabetes in Chinese Hans from Shanghai. J Hum Genet. 2006;51(7):605-10.
41. Yang S, Zhao HL, Xu KF, Qian Y, Wu M, Yang T, Chen YC, Zhao XH, Chen JF, Wen JB, et al. Evaluation of common variants in MG53 and the risk of type 2 diabetes and insulin resistance in Han Chinese. SpringerPlus. 2016;5:11.
42. Tang L, Tong Y, Cao H, Xie S, Yang Q, Zhang F, Zhu Q, Huang L, Lu Q, Yang Y, et al. The MTMR9 rs2293855 polymorphism is associated with glucose tolerance, insulin secretion, insulin sensitivity and increased risk of prediabetes. Gene. 2014;546(2):150-5.
43. Ruchat SM, Girard M, Weisnagel SJ, Bouchard C, Vohl MC, Perusse L. Association between mu-opioid receptor-1 102T > C polymorphism and intermediate type 2 diabetes phenotypes: Results from the Quebec Family Study (QFS). Clin Exp Pharmacol Physiol. 2008;35(9):1018-22.
44. Heni M, Haupt A, Schafer SA, Ketterer C, Thamer C, Machicao F, Stefan N, Staiger H, Haring HU, Fritsche A. Association of obesity risk SNPs in PCSK1 with insulin sensitivity and proinsulin conversion. Bmc Medical Genetics. 2010;11:8.
45. Chang TJ, Chiu YF, Sheu WHH, Shih KC, Hwu CM, Quertermous T, Jou YS, Kuo SS, Chang YC, Chuang LM. Genetic polymorphisms of PCSK2 are associated with glucose homeostasis and progression to type 2 diabetes in a Chinese population. Sci Rep. 2015;5:9.
46. Oberkofler H, Linnemayr V, Weitgasser R, Klein K, Xie MQ, Iglseder B, Krempler F, Paulweber B, Patsch W. Complex haplotypes of the PGC-1 alpha gene are associated with carbohydrate metabolism and type 2 diabetes. Diabetes. 2004;53(5):1385-93.
47. Kachele M, Hennige AM, Machann J, Hieronimus A, Lamprinou A, Machicao F, Schick F, Fritsche A, Stefan N, Nurnberg B, et al. Variation in the Phosphoinositide 3-Kinase Gamma Gene Affects Plasma HDL-Cholesterol without Modification of Metabolic or Inflammatory Markers. PLoS One. 2015;10(12):17.
48. Yan J, Hu C, Jiang F, Zhang R, Wang J, Tang S, Peng D, Chen M, Bao Y, Jia W. Genetic variants of PLA2G6 are associated with Type 2 diabetes mellitus and triglyceride levels in a Chinese population. Diabetic Medicine. 2015;32(2):280-6.
49. Bosse Y, Weisnagel SJ, Bouchard C, Despres JP, Perusse L, Vohl MC. Combined effects of PPAR gamma(2) P12A and PPAR alpha L162V polymorphisms on glucose and insulin homeostasis: The Quebec Family Study. J Hum Genet. 2003;48(12):614-21.
50. Ruchat SM, Weisnagel SJ, Vohl MC, Rankinen T, Bouchard C, Perusse L. Evidence for Interaction between PPARG Pro12Ala and PPARGC1A Gly482Ser Polymorphisms in Determining Type 2 Diabetes Intermediate Phenotypes in Overweight Subjects. Exp Clin Endocrinol Diabet. 2009;117(9):455-9.
51. Hansen TH, Vestergaard H, Jorgensen T, Jorgensen ME, Lauritzen T, Brandslund I, Christensen C, Pedersen O, Hansen T, Gjesing AP. Impact of PTBP1 rs11085226 on glucose-stimulated insulin release in adult Danes. Bmc Medical Genetics. 2015;16:9.

52. Lindner I, Helwig U, Rubin D, Fischer A, Marten B, Schreiber S, Doring F, Schrezenmeir J. Prostaglandin E synthase 2 (PTGES2) Arg298His polymorphism and parameters of the metabolic syndrome. Mol Nutr Food Res. 2007;51(12):1447-51.
53. Torekov SS, Larsen LH, Glumer C, Borch-Johnsen Y, Jorgensen T, Holst JJ, Madsen OD, Hansen T, Pedersen O. Evidence of an association between the Arg72 allele of the peptide YY and increased risk of type 2 diabetes. Diabetes. 2005;54(7):2261-5.
54. Burgdorf KS, Gjesing AP, Grarup N, Justesen JM, Sandholt CH, Witte DR, Jorgensen T, Madsbad S, Hansen T, Pedersen O. Association studies of novel obesity-related gene variants with quantitative metabolic phenotypes in a population-based sample of 6,039 Danish individuals. Diabetologia. 2012;55(1):105-13.
55. Bouchard L, Weisnagel SJ, Engert JC, Hudson TJ, Bouchard C, Vohl MC, Perusse L. Human resistin gene polymorphism is associated with visceral obesity and fasting and oral glucose stimulated C-peptide in the Quebec Family Study. J Endocrinol Invest. 2004;27(11):1003-9.
56. Enigk U, Breitfeld J, Schleinitz D, Dietrich K, Halbritter J, Fischer-Rosinsky A, Enigk B, Muller I, Spranger J, Pfeiffer A, et al. Role of genetic variation in the human sodium-glucose cotransporter 2 gene (SGLT2) in glucose homeostasis. Pharmacogenomics. 2011;12(8):1119-26.
57. Bouatia-Naji N, Vatin V, Lecoeur C, Heude B, Proenca C, Veslot J, Jouret B, Tichet J, Charpentier G, Marre M, et al. Secretory granule neuroendocrine protein 1 (SGNE1) genetic variation and glucose intolerance in severe childhood and adult obesity. Bmc Medical Genetics. 2007;8:9.
58. Capuano MM, Sorkin JD, Chang YP, Ling H, O'Connell JR, Rothman PB, Mitchell BD, Silver KD. Polymorphisms in the SOCS7 gene and glucose homeostasis traits. BMC Res Notes. 2013;6:235.
59. Weisnagel SJ, Rankinen T, Nadeau A, Rao DC, Chagnon YC, Perusse L, Bouchard C. Decreased fasting and oral glucose stimulated C-peptide in nondiabetic subjects with sequence variants in the sulfonylurea receptor 1 gene. Diabetes. 2001;50(3):697-702.
60. Ketterer C, Mussig K, Heni M, Dudziak K, Randrianarisoa E, Wagner R, Machicao F, Stefan N, Holst JJ, Fritsche A, et al. Genetic variation within the TRPM5 locus associates with prediabetic phenotypes in subjects at increased risk for type 2 diabetes. Metab-Clin Exp. 2011;60(9):1325-33.
61. Halsall DJ, Luan J, Saker P, Huxtable S, Farooqi IS, Keogh J, Wareham NJ, O'Rahilly S. Uncoupling protein 3 genetic variants in human obesity: the c-55t promoter polymorphism is negatively correlated with body mass index in a UK Caucasian population. Int J Obes Relat Metab Disord. 2001;25(4):472-7.
62. Putt W, Palmen J, Tahri-Daizadeh N, Flavell DM, Humphries SE, Talmud PJ, Grp E. Variation in USF1 shows haplotype effects, gene : gene and gene : environment associations with glucose and lipid parameters in the European Atherosclerosis Research Study II. Hum Mol Genet. 2004;13(15):1587-97.
63. Jian WX, Luo TH, Gu YY, Zhang HL, Zheng S, Dai M, Han JF, Zhao Y, Li G, Luo M. The visfatin gene is associated with glucose and lipid metabolism in a Chinese population. Diabet Med. 2006;23(9):967-73.
64. Chiu KC, Chuang LM, Yoon C. The vitamin D receptor polymorphism in the translation initiation codon is a risk factor for insulin resistance in glucose tolerant Caucasians. BMC Medical Genetics. 2001;2 (no pagination).
