# Supplementary material for: Association between Genotype and the Glycemic Response to an Oral Glucose Tolerance Test: A Systematic Review
Source: Nutrients. 2023 Mar 30;15(7):1695. doi: 10.3390/nu15071695 (PMC10096950; doi:10.3390/nu15071695)
Supplement: Supplementary file 1 [file nutrients-15-01695-s001.zip › Table S2.pdf]

Contact Info: Christina Holzapfel, PhD, Institute for Nutritional Medicine, School of Medicine, Technical University of Munich, Georg-Brauchle-Ring 62, 80992 Munich. E-Mail: christina.holzapfel@tum.de; Phone: 0049 89 289 249 23; Fax: 0049 89 289 249 22

Supplementary Table S2. Associations between SNPs and gAUC after an OGTT in adults. Gene loci are examined in two different articles.

Study characteristics	SNP	Sample size used	Results (*p*-Value)*	Reference
ADRB2				
Sedentary, postmenopausal white and black women, aged 45–80 years, non-smokers, no previous diagnosis of diabetes or cardiovascular disease, obesity (> 35% body fat), living in the Maryland area	rs1042713	123<br>Participants without diabetes or treatment-naive participants	0.004[1,7]<br>Significant difference between homozygous and heterozygous carriers of the minor allele (A) and the wild type with heterozygous carriers having the lowest gAUC	[1]
	rs1042714	123<br>Participants without diabetes or treatment-naive participants	n. s.[1,7]	
40 families with obesity but without diabetes, genetic trait of obesity, Caucasian origin		16<br>Men without diabetes and obesity	0.988[2,7]	[2]
			0.710[3,7]	
		22<br>Men without diabetes but with obesity	0.225[2,7]	
			0.089[3,7]	
ADRB3				
40 families with obesity but without diabetes, genetic trait of obesity, Caucasian origin	rs4994	16<br>Men without diabetes and obesity	0.013[2,7]<br>Significant difference between heterozygous carriers of the minor allele (G) and the wild type	[2]
Population-based study, elderly same-sex Danish twins, Caucasian decent		20<br>Dizygotic twins without diabetes	0.92[3,7]	[3]
ApoB				
Population-based study, elderly same-sex Danish twins, Caucasian decent	rs693	548<br>Participants without diabetes or treatment-naive participants	n. s.[1,8,24]	[4]
	I711T	564<br>Participants without diabetes or treatment-naive participants	n. s.[1,9,24]	[5]
	A591V		n. s.[1,9,24]	
	L2712P		n. s.[1,9,24]	
	R3611Q		n. s.[1,9,24]	

Study characteristics	SNP	Sample size used	Results (*p*-Value)*	Reference
	E4154K		n. s.[1,9,24]	
CAPN10				
Essential hypertensive, unrelated participants without diabetes from the Chinese Han population	rs2975760	961<br>Participants without diabetes	n. s.[1,10,26]	[6]
			n. s.[4,10,26]	
			n. s.[5,10,26]	
Non-diabetic offsprings of patients with T2DM from Finland	rs2975760	158<br>Participants without diabetes	n. s.[1,11]	[7]
**METSIM**<br>Finnish men, aged 50-70 years, randomly selected from the population register of Kuopio		234<br>Participants (with a history of T2DM in first-degree relatives) without diabetes and obesity	n. s.[1,12]	
Essential hypertensive, unrelated participants without diabetes from the Chinese Han population	rs3792267	961<br>Participants without diabetes	n. s.[1,10,26]	[6]
			n. s.[4,10,26]	
			n. s.[5,10,26]	
Non-diabetic offsprings of patients with T2DM from Finland		71<br>Participants without diabetes	0.114[1,11]	[7]
**METSIM**<br>Finnish men, aged 50-70 years, randomly selected from the population register of Kuopio		234<br>Participants (with a history of T2DM in first-degree relatives) without diabetes and obesity	0.564[1,12]	
Non-diabetic offsprings of patients with T2DM from Finland	rs5030952	158<br>Participants without diabetes	n. s.[1,11]	
**METSIM**<br>Finnish men, aged 50-70 years, randomly selected from the population register of Kuopio		234<br>Participants (with a history of T2DM in first-degree relatives) without diabetes and obesity	n. s.[1,12]	
DIO2				
**Amish Family Diabetes Study**<br>Old Order Amish population, participants with previously T2DM and first- and second-degree relatives, aged ≥ 18 years	rs225014	747<br>Participants without diabetes	0.34[1,13,25]	[8]
**Inter99**<br>Population-based study, participants aged 30-60 years, Caucasian decent		5,843<br>Participants without diabetes or treatment-naive participants	0.6[1,13]	[9]
			0.4[4,14]	
			0.9[5,14]	
FABP2				
**EARS II**	rs1799883	666	0.6[1,15]	[10]

Study characteristics	SNP	Sample size used	Results (*p*-Value)*	Reference
European men, aged 18-28 years, cases with a family history of premature acute myocardial infarction before the age of 55 years and controls with close birth date to the case	rs1799883	Participants without diabetes		
Healthy participants, aged 50-75 years, sedentary lifestyle, non-smoking, BMI < 37 kg/m^2		122<br>Participants without diabetes or treatment-naive participants	0.43[4,16]	[11]
		120<br>Participants without diabetes	n. s.[4,16]	
		36<br>Participants (also underwent an oral lipid tolerance test) without diabetes	n. s.[4,16]	
FTO				
**Quebec Family Study**<br>French-Canadian families (phase 1: randomly selected; phase 2/3: at least one person with obesity per family) living in and around the Quebec city area	rs1421085	908<br>Participants without diabetes	0.26[1,13,25]	[12]
			0.57[5,13,25]	
	rs17817449		0.44[1,13,25]	
			0.51[5,13,25]	
Non-smoking participants without diabetes, aged ≥ 65 years, BMI ≥ 30 kg/m^2, sedentary lifestyle; ethnicity: non-Hispanic Whites, Hispanic Whites, African American and other	rs8050136	165<br>Participants without diabetes	0.19[4,17]	[13]
HNF-1α				
Unrelated, healthy, normotensive, glucose-tolerant participants; ethnicity: Caucasian, Asian American, Mexican American, African American	rs1169288	52<br>Normoglycemic participants	n. s.[1,18]	[14]
German family study with family history of T2DM, obesity or dyslipoproteinaemia, living in Dresden and surrounding areas		1,479<br>Participants without diabetes	0.564[1,19]	[15]
		616<br>Normoglycemic participants	n. s.[1,19]	
		652<br>Participants with impaired glucose tolerance and/or impaired fasting glucose	n. s.[1,19]	
	rs1800574	1,479<br>Participants without diabetes	0.173[2,19]	
		616<br>Normoglycemic participants	n. s.[2,19]	
		652<br>Participants with impaired glucose tolerance and/or impaired fasting glucose	n. s.[2,19]	
INS-VNTR				
**Inter99**<br>Population-based study, participants aged 30-60 years, Caucasian decent	rs689	4,444<br>Normoglycemic participants	0.24[1,14]	[16]
			0.12[5,14]	

Study characteristics	SNP	Sample size used	Results (*p*-Value)*	Reference
			0.80[4,14]	
**Inter99**<br>Population-based study, participants aged 30-60 years, Caucasian decent	rs689	490<br>Participants with impaired fasting glucose	0.30[1,14]	[16]
			0.32[5,14]	
			0.15[4,14]	
		678<br>Participants with impaired glucose tolerance	0.57[1,14]	
			0.29[5,14]	
			0.77[4,14]	
		5,612<br>Participants without diabetes	n. s.[1,14]	
			n. s.[5,14]	
			n. s.[4,14]	
**EARS II**<br>European men, aged 18-28 years, cases with a family history of premature acute myocardial infarction before the age of 55 years and controls with close birth date to the case		822<br>Participants without diabetes	n. s.[1,20]	[17]
IRS-1				
Asian Indians and Caucasians without diabetes	rs1801278	158<br>Asian Indians without diabetes	n. s.[4,7]	[18]
		152<br>Caucasians without diabetes	n. s.[4,7]	
**EARS II**<br>European men, aged 18-28 years, cases with a family history of premature acute myocardial infarction before the age of 55 years and controls with close birth date to the case	rs2943641	714<br>Participants without diabetes	0.43[4,20]	[19]
MNTR1B				
**PPP-Botnia Study**<br>Randomly selected Caucasians, aged 17-78 years, living in Western Finland	rs10830963	4,455<br>Participants without diabetes	< 0.001[1,21]<br>Significant difference between homozygous and heterozygous carriers of the minor allele (G) and the wild type; the addition of each copy of the minor allele was associated with higher gAUC	[20]
		3,422<br>Participants without diabetes	< 0.001[1,22]<br>Significant difference between homozygous and heterozygous carriers of the minor allele (G) and the wild type; the addition of each copy of the minor allele was associated with higher gAUC	[21]
MTP				
**MICK**<br>European men, aged 45-65 years, with residency near Kiel	rs3816873	721<br>Participants without diabetes	n. s.[4,7]	[22]

Study characteristics	SNP	Sample size used	Results (*p*-Value)*	Reference
Sedentary, non-smoking men with adiposity and visceral adipose tissue accumulation, living in the Quebec area	rs1800591	227<br>Participants without diabetes	0.64[4,7]	[23]
PON1				
Healthy, glucose-tolerant Whites	rs662	84<br>Participants without diabetes	n. s.[1,7]	[24]
**EARS II**<br>European men, aged 18-28 years, cases with a family history of premature acute myocardial infarction before the age of 55 years and controls with close birth date to the case		775<br>Participants without diabetes	n. s.[1,23]	[25]
			n. s.[5,23]	
Healthy, glucose-tolerant Whites	rs854560	84<br>Participants without diabetes	n. s.[1,7]	[24]
**EARS II**<br>European men, aged 18-28 years, cases with a family history of premature acute myocardial infarction before the age of 55 years and controls with close birth date to the case		775<br>Participants without diabetes	0.013[1,23]<br>Significant difference between homozygous and heterozygous carriers of the minor allele (T) and the wild type	[25]
			< 0.004[5,23]<br>Significantly lower gAUC in carriers of the minor allele (T) compared to the wild type	
SLC2A10				
**Inter99**<br>Population-based study, participants aged 30-60 years, Caucasian decent	rs2235491	4,372<br>Normoglycemic participants	0.2[5,14]	[26]
			0.5[1,14]	
		1,264<br>Participants aged ≥ 50 years and without diabetes	n. s.[5,14]	
			n. s.[1,14]	
Unrelated, glucose tolerant Europeans		507<br>Normoglycemic participants	0.8[5,14]	[27]
UCP1				
40 families with obesity but without diabetes, genetic trait of obesity, Caucasian origin	rs1800592	118<br>Participants without diabetes	n. s.[2,7]	[28]
			n. s.[6,7]	
			n. s.[3,7]	
		122<br>Participants without diabetes	n. s.[1,7]	[29]

ADRB2, Adrenoceptor Beta 2; ADRB3, Adrenoceptor Beta 3; ApoB, Apolipoprotein B; BMI, body mass index; *CAPN10, Calpain 10; DIO2, Iodothyronine Deiodinase 2;* EARS II, European Atherosclerosis Research Study; *FABP2, Fatty Acid Binding Protein 2*; *FTO, Fat Mass And Obesity-Associated;* gAUC, glucose area under the curve; *HNF-1α, HNF1 Homeobox A*; *INS VNTR, Insulin Variable Number Of Tandem Repeats;* Inter99, Lifestyle Intervention in a General Population for Prevention of Ischaemic Heart Disease; *IRS-1, Insulin Receptor Substrate 1;* METSIM,

Metabolic Syndrome in Men; MICK, Metabolic Intervention Cohort Kiel; *MNTR1B, Melatonin Receptor 1B; MTP, Microsomal Triglyceride Transfer Protein*; n. s., not significant; OGTT, oral glucose tolerance test; *PON1, Paraoxonase 1;* PPP-Botnia Study, Prevalence, Prediction and Prevention of diabetes-Botnia Study; rs, reference SNP; *SLC2A10, Solute Carrier Family 2 Member 10;* SNP, single nucleotide polymorphism; T2DM, type 2 diabetes mellitus; *UCP1, Uncoupling Protein 1*; * p-value as indicated in the report; [1] Additive genetic model; [2] gAUC between heterozygous carriers of the minor allele and the wild type were compared; [3] gAUC between homozygous carriers of the minor allele and the wild type were compared; [4] Dominant genetic model; [5] Recessive genetic model; [6] gAUC between homozygous carriers and heterozygous carriers of the minor allele were compared; [7] No further information about adjustment; [8] Adjusted for gender, age, glucose tolerance status; [9] Adjusted for gender, age, BMI, glucose tolerance status; [10] Adjusted for age, sex, BMI, 24h mean systolic blood pressure, 24h mean diastolic blood pressure, ACEI/ARB treatment; [11] Adjusted for age, BMI, family relationship; [12] Adjusted for age, BMI; [13] Adjusted for age, sex; [14] Adjusted for age, sex, BMI; [15] Adjusted for fasting values; [16] Adjusted for age, percentage of body fat; [17] Adjusted for age, gender, race; [18] Adjusted for age, BMI, waist/hip ratio, systolic and diastolic blood pressure, gender, ethnicity; [19] Adjusted for age, BMI, systolic and diastolic blood pressure; [20] Adjusted for age, centre, case/control status; [21] Adjusted for sex, age, BMI, education, current smoking status, alcohol consumption, physical activity, season, depressive symptoms; [22] Adjusted for sex, age, BMI, smoking, alcohol use, physical activity, education at baseline; [23] Adjusted for age, centre, case/control status, fasting glucose; [24] Random effect of twin pairs; [25] Non-independency of family members was statistically taken into account; [26] Bonferroni-correction applied.

References

1. Prior SJ, Goldberg AP, Ryan AS. ADRB2 haplotype is associated with glucose tolerance and insulin sensitivity in obese postmenopausal women. Obesity (Silver Spring). 2011;19(2):396-401.
2. Malczewska-Malec M, Wybranska I, Leszczynska-Golabek I, Niedbal S, Kwasniak M, Hartwich J, Kiec-Wilk B, Motyka M, Szopa M, Dembinska-Kiec A. An analysis of the link between polymorphisms of the beta2 and beta3 adrenergic receptor gene and metabolic parameters among Polish Caucasians with familial obesity. Medical Science Monitor. 2003;9(6):CR225-CR34.
3. Hojlund K, Christiansen C, Bjornsbo KS, Poulsen P, Bathum L, Henriksen JE, Lammert O, Beck-Nielsen H. Energy expenditure, body composition and insulin response to glucose in male twins discordant for the Trp64Arg polymorphism of the beta(3)-adrenergic receptor gene. Diabetes Obes Metab. 2006;8(3):322-30.
4. Bentzen J, Poulsen P, Vaag A, Beck-Nielsen H, Fenger M. The influence of the polymorphism in apolipoprotein B codon 2488 on insulin and lipid levels in a Danish twin population. Diabet Med. 2002;19(1):12-8.
5. Bentzen J, Poulsen P, Vaag A, Fenger M. Further studies of the influence of apolipoprotein B alleles on glucose and lipid metabolism. Hum Biol. 2003;75(5):687-703.
6. Zhou XO, Wang Y, Zhang Y, Gao PJ, Zhu DL. Association of CAPN10 gene with insulin sensitivity, glucose tolerance and renal function in essential hypertensive patients. Clin Chim Acta. 2010;411(15-16):1126-31.
7. Pihlajamaki J, Salmenniemi U, Vanttinen M, Ruotsalainen E, Kuusisto J, Vauhkonen I, Kainulainen S, Ng MC, Cox NJ, Bell GI, et al. Common polymorphisms of calpain-10 are associated with abdominal obesity in subjects at high risk of type 2 diabetes. Diabetologia. 2006;49(7):1560-6.
8. Mentuccia D, Thomas MJ, Coppotelli G, Reinhart LJ, Mitchell BD, Shuldiner AR, Celi FS. The Thr92Ala deiodinase type 2 (DIO2) variant is not associated with type 2 diabetes or indices of insulin resistance in the old order of Amish. Thyroid. 2005;15(11):1223-7.

9. Grarup N, Andersen MK, Andreasen CH, Albrechtsen A, Borch-Johnsen K, Jorgensen T, Auwerx J, Schmitz O, Hansen T, Pedersen O. Studies of the common DIO2 Thr92Ala polymorphism and metabolic phenotypes in 7342 Danish white subjects. J Clin Endocrinol Metab. 2007;92(1):363-6.
10. Tahvanainen E, Molin M, Vainio S, Tiret L, Nicaud V, Farinaro E, Masana L, Enholm C. Intestinal fatty acid binding protein polymorphism at codon 54 is not associated with postprandial responses to fat and glucose tolerance tests in healthy young Europeans. Results from EARS II participants. Atherosclerosis. 2000;152(2):317-25.
11. Weiss EP, Brandauer J, Kulaputana O, Ghiu IA, Wohn CR, Phares DA, Shuldiner AR, Hagberg JM. FABP2 Ala54Thr genotype is associated with glucoregulatory function and lipid oxidation after a high-fat meal in sedentary nondiabetic men and women. Am J Clin Nutr. 2007;85(1):102-8.
12. Do R, Bailey SD, Desbiens K, Belisle A, Montpetit A, Bouchard C, Perusse L, Vohl MC, Engert JC. Genetic variants of FTO influence adiposity, insulin sensitivity, leptin levels, and resting metabolic rate in the Quebec Family Study. Diabetes. 2008;57(4):1147-50.
13. Armamento-Villareal R, Wingkun N, Aguirre LE, Kulkarny V, Napoli N, Colleluori G, Qualls C, Villareal DT. The FTO gene is associated with a paradoxically favorable cardiometabolic risk profile in frail, obese older adults. Pharmacogenet Genomics. 2016;26(4):154-60.
14. Chiu KC, Chuang LM, Yoon C. The vitamin D receptor polymorphism in the translation initiation codon is a risk factor for insulin resistance in glucose tolerant Caucasians. BMC Medical Genetics. 2001;2 (no pagination).
15. Bergmann A, Li J, Selisko T, Reimann M, Fischer S, Grassler J, Schulze J, Bornstein SR, Schwarz PEH. The A98V Single Nucleotide Polymorphism (SNP) in Hepatic Nuclear Factor 1 alpha (HNF-1 alpha) is Associated with Insulin Sensitivity and beta-Cell Function. Exp Clin Endocrinol Diabet. 2008;116:S50-S5.
16. Hansen SK, Gjesing AP, Rasmussen SK, Glumer C, Urhammer SA, Andersen G, Rose CS, Drivsholm T, Torekov SK, Jensen DP, et al. Large-scale studies of the HphI insulin gene variable-number-of-tandem-repeats polymorphism in relation to Type 2 diabetes mellitus and insulin release. Diabetologia. 2004;47(6):1079-87.
17. Waterworth DM, Jansen H, Nicaud V, Humphries SE, Talmud PJ, grp Es. Interaction between insulin (VNTR) and hepatic lipase (LIPC-514C > T) variants on the response to an oral glucose tolerance test in the EARSII group of young healthy men. Biochim Biophys Acta-Mol Basis Dis. 2005;1740(3):375-81.
18. Abate N, Carulli L, Cabo-Chan Jr A, Chandalia M, Snell PG, Grundy SM. Genetic Polymorphism PC-1 K121Q and Ethnic Susceptibility to Insulin Resistance. Journal of Clinical Endocrinology and Metabolism. 2003;88(12):5927-34.
19. Yiannakouris N, Cooper JA, Shah S, Drenos F, Ireland HA, Stephens JW, Li KW, Elkeles R, Godsland IF, Kivimaki M, et al. IRS1 gene variants, dysglycaemic metabolic changes and type-2 diabetes risk. Nutr Metab Carbiovasc Dis. 2012;22(12):1024-30.
20. Haljas K, Lahti J, Tuomi T, Isomaa B, Eriksson JG, Groop L, Raikkonen K. Melatonin receptor 1B gene rs10830963 polymorphism, depressive symptoms and glycaemic traits. Ann Med. 2018;50(8):704-12.
21. Haljas K, Hakaste L, Lahti J, Isomaa B, Groop L, Tuomi T, Raikkonen K. The associations of daylight and melatonin receptor 1B gene rs10830963 variant with glycemic traits: the prospective PPP-Botnia study. Ann Med. 2019;51(1):58-67.
22. Rubin D, Helwig U, Pfeuffer M, Schreiber S, Boeing H, Fisher E, Pfeiffer A, Freitag-Wolf S, Foelsch UR, Doering F, et al. A common functional exon polymorphism in the microsomal triglyceride transfer protein gene is associated with type 2 diabetes, impaired glucose metabolism and insulin levels. J Hum Genet. 2006;51(6):567-74.
23. St-Pierre J, Lemieux I, Miller-Felix I, Prud'homme D, Bergeron J, Gaudet D, Nadeau A, Despres JP, Vohl MC. Visceral obesity and hyperinsulinemia modulate the impact of the microsomal triglyceride transfer protein -493G/T polymorphism on plasma lipoprotein levels in men. Atherosclerosis. 2002;160(2):317-24.
24. Chiu KC, Chuang LM, Chu A, Lu J, Hu J, Fernando S. Association of paraoxonase 1 polymorphism with beta-cell function - A case of molecular heterosis. Pancreas. 2004;28(4):E96-E103.
25. Deakin S, Leviev I, Nicaud V, Meynet MCB, Tiret L, James RW, European Atherosclerosis Risk S. Paraoxonase-1 L55M polymorphism is associated with an abnormal oral glucose tolerance test and differentiates high risk coronary disease families. J Clin Endocrinol Metab. 2002;87(3):1268-73.
26. Rose CS, Andersen G, Hamid YH, Glumer C, Drivsholm T, Borch-Johnsen K, Jorgensen T, Pedersen O, Hansen T. Studies of relationships between the GLUT10 Ala206Thr polymorphism and impaired insulin secretion. Diabetic Medicine. 2005;22(7):946-9.
27. Andersen G, Rose CS, Hamid YH, Drivsholm T, Borch-Johnsen K, Hansen T, Pedersen O. Genetic variation of the GLUT10 glucose transporter (SLC2A10) and relationships to type 2 diabetes and intermediary traits. Diabetes. 2003;52(9):2445-8.
28. Kiec-Wilk B, Wybranska I, Malczewska-Malec M, Leszczynska-Golabek I, Partyka L, Niedbal S, Jabrocka A, Dembinska-Kiec A. Correlation of the-3826A > G polymorphism in the promoter of the uncoupling protein 1 gene with obesity and metabolic disorders in obese families from southern Poland. J Physiol Pharmacol. 2002;53(3):477-90.
29. Maiczewska-Malec M, Wybranska I, Leszczynska-Golabek I, Partyka L, Hartwich J, Jabrocka A, Kiec-Wilk B, Kwasniak M, Motyka M, Dembinska-Kiec A. Analysis of candidate genes in Polish families with obesity. Clin Chem Lab Med. 2004;42(5):487-93.
